# Multiomics characterization of pyroptosis in the tumor microenvironment and therapeutic relevance in metastatic melanoma

**DOI:** 10.1186/s12916-023-03175-0

**Published:** 2024-01-17

**Authors:** Wenqiong Chen, Yi He, Guowei Zhou, Xiang Chen, Youqiong Ye, Guanxiong Zhang, Hong Liu

**Affiliations:** 1 The Department of Dermatology, Xiangya Hospital, Central South University, Changsha, China; 2 Hunan Key Laboratory of Skin Cancer and Psoriasis, Hunan Engineering Research Center of Skin Health and Disease, Xiangya Hospital, Changsha, China; 3 National Clinical Research Center for Geriatric Disorders, Xiangya Hospital, Changsha, China; 4 Xiangya Clinical Research Center for Cancer Immunotherapy, Central South University, Changsha, China; 5 National Engineering Research Center of Personalized Diagnostic and Therapeutic Technology, Changsha, China; 6 Furong Laboratory, Changsha, Hunan China; 7 Department of Immunology and Microbiology, Shanghai Institute of Immunology, Shanghai Jiao Tong University School of Medicine, Shanghai, 200025 China; 8 Research Center of Molecular Metabolomics, Xiangya Hospital, Central South University, Changsha, China; 9 Big Data Institute, Central South University, Changsha, 410083 China

**Keywords:** Pyroptosis, Metastatic melanoma, Tumor microenvironment, Immunotherapy, Multiomics

## Abstract

**Background:**

Pyroptosis, mediated by gasdermins with the release of multiple inflammatory cytokines, has emerged as playing an important role in targeted therapy and immunotherapy due to its effectiveness at inhibiting tumor growth. Melanoma is one of the most commonly used models for immunotherapy development, though an inadequate immune response can occur. Moreover, the development of pyroptosis-related therapy and combinations with other therapeutic strategies is limited due to insufficient understanding of the role of pyroptosis in the context of different tumor immune microenvironments (TMEs).

**Methods:**

Here, we present a computational model (pyroptosis-related gene score, PScore) to assess the pyroptosis status. We applied PScore to 1388 melanoma samples in our in-house cohort and eight other publicly available independent cohorts and then calculated its prognostic power of and potential as a predictive marker of immunotherapy efficacy. Furthermore, we performed association analysis for PScore and the characteristics of the TME by using bulk, single-cell, and spatial transcriptomics and assessed the association of PScore with mutation status, which contributes to targeted therapy.

**Results:**

Pyroptosis-related genes (PRGs) showed distinct expression patterns and prognostic predictive ability in melanoma. Most PRGs were associated with better survival in metastatic melanoma. Our PScore model based on genes associated with prognosis exhibits robust performance in survival prediction in multiple metastatic melanoma cohorts. We also found PScore to be associated with *BRAF* mutation and correlate positively with multiple molecular signatures, such as KRAS signaling and the IFN gamma response pathway. Based on our data, melanoma with an immune-enriched TME had a higher PScore than melanoma with an immune-depleted or fibrotic TME. Additionally, monocytes had the highest PScore and malignant cells and fibroblasts the lowest PScore based on single-cell and spatial transcriptome analyses. Finally, a higher PScore was associated with better therapeutic efficacy of immune checkpoint blockade, suggesting the potential of pyroptosis to serve as a marker of immunotherapy response.

**Conclusions:**

Collectively, our findings indicate that pyroptosis is a prognostic factor and is associated with the immune response in metastatic melanoma, as based on multiomics data. Our results provide a theoretical basis for drug combination and reveal potential immunotherapy response markers.

**Supplementary Information:**

The online version contains supplementary material available at 10.1186/s12916-023-03175-0.

## Background

Malignant melanoma is the most aggressive skin cancer, with poor prognosis. Recently, rapid development of immunotherapies and targeted therapies has enabled significant improvements in overall survival (OS) and disease-free survival as well as impressive response rates in melanoma [1–3]. However, only 19–45% of patients respond to these therapeutic modalities, and some patients may even experience relapse [2, 3]. Accordingly, there is an urgent need to elucidate the molecular mechanisms underlying melanoma pathogenesis to help discover novel therapeutic strategies for improving the efficacy of cancer treatment.

Pyroptosis, a type of regulated cell death executed by the gasdermin (GSDM) protein family that is accompanied by cell membrane pore formation and IL18/IL1β release, plays an essential role in the immune response [4–6]. As pyroptotic cells release inflammatory factors, damaged plasma membranes induce chemokine production and recruit a variety of immune cells [7, 8]. Studies have shown that pyroptosis results in amplified cellular immunity, as toxic lymphocytes, including natural killer (NK) and CD8 + T cells, release granzymes such as GZMA and GZMB to cleave GSDMB and GSDME, respectively [9–11]. A recent study has shown that inducing breast tumor cell pyroptosis with trimethylamine N-oxide (TMAO) promotes antitumor immunity [12]. It has also been reported that pyroptosis of a few tumor cells is sufficient to enhance antitumor immunity and synergize with immune checkpoint therapy in a 4T1 tumor model [13]. BRAF and MEK inhibitor combinations induce GSDME-dependent pyroptosis, and resistant cells do not acquire drug sensitivity unless GSDME cleavage and pyroptosis are reinduced [14]. Furthermore, the key molecule of pyroptosis NLRP3 is required for the TH2 cell transcriptome program in CD4( +) T cells, and NLRP3 deficiency promotes melanoma growth [15]. Paradoxically, NLRP3 plays an immunosuppressive role in melanoma tumor cells by recruiting myeloid-derived suppressor cells (MDSCs) [16, 17]. In addition, in vivo experiments show that inhibiting *GSDMC* transcription and thereby suppressing pyroptosis alleviates tumor necrosis symptoms and prolongs the survival of tumor-bearing mice [18]. These results suggest that pyroptosis is a double-edged sword in tumors and point to the importance of the executor involved and the cell type in which the process occurs. Overall, there is a lack of in-depth research on the role pyroptosis plays in melanoma.

There have been several attempts thus far to establish prognostic models related to pyroptosis [19–29]. The majority of them have been built based on least absolute shrinkage and selection operator (LASSO) regression [30] with distinct gene sets as input. Previous studies only included some pyroptosis-related genes (PRGs), which may lead to loss of information. In addition, due to the nature of LASSO regression, these models more reflect efficacy for prognosis than the state of pyroptosis itself. Thus, the status and heterogeneity of pyroptosis in melanoma determined by the expression pattern of all PRGs remains unclear.

In this study, we evaluated pyroptosis status using single-sample gene set enrichment analysis (ssGSEA), followed by comprehensive analysis to understand the molecular alterations and biological effects of pyroptosis. We observed that pyroptosis can act as an independent prognostic factor and validated it using eight independent datasets. We found distinct roles for pyroptosis in primary and metastatic melanoma patients, with a certain association between pyroptosis and *BRAF* mutation status, revealing a potential combination strategy of drug usage in melanoma. Functionally, pyroptosis levels correlated positively with multiple immune response-related pathways but negatively with carcinogenesis-related pathways. Mechanistically, we investigated the association between pyroptosis level and immune or DNA damage features. Then, we assessed the heterogeneity in pyroptosis status among different cell subtypes in the tumor immune microenvironment (TME) at bulk, single-cell, and spatial transcriptome levels. Finally, we determined the therapeutic value of different pyroptosis statuses in immunotherapy.

## Methods

### Data collection and processing

Multiomics data and clinical data for cutaneous melanoma (CM) were downloaded from The Cancer Genome Atlas (TCGA) data portal (https://portal.gdc.cancer.gov/). Independent melanoma cohorts with or without immunotherapy were downloaded from Gene-Expression Omnibus (GEO), Sequence Read Archive (SRA), and Database of Genotypes and Phenotypes (dbGaP) (Additional file 1: Table S1). In-house data with immunotherapy were downloaded from Table S9 of our previous study [31]. Melanoma patients with anti-PD1 treatment were collected from Xiangya Hospital and Fudan University Shanghai Cancer Center, and the study was approved by our hospital ethics committee (Committee number: 202103213).

Processed single-cell transcriptome data for melanoma were retrieved from TISCH (http://tisch.comp-genomics.org/) under accession numbers GSE72056 (4645 single cells including malignant, immune, stromal, and endothelial cells from 19 melanoma samples) and GSE115978 (7186 single cells including malignant, immune, stromal, and endothelial cells from 33 melanoma samples). We extracted cells belonging to metastatic patients by using the “subset” function. Spatial transcriptome data of melanoma were obtained from the BayesSpace package [32, 33]. Processed proteomics data and clinical data of melanoma treated with anti-PD1 were obtained from Harel and Beck et al. [34, 35].

### Construction of the pyroptosis-related gene score (PScore) model to estimate pyroptosis status in melanoma

We collected 75 PRGs from publications [4, 5, 9, 10, 36–81] and Molecular Signatures Database (MSigDB, https://www.gsea-msigdb.org/gsea/msigdb) [82–84], and 74 of them were retained for analysis (Table 1). Specifically, we collected 62 PRGs from the published literature [4, 5, 9, 10, 36–81] (Table 1, colored green) and supplemented them with gene sets from MSigDB, including “GOBP_PYROPTOSIS.v2023.1.Hs.grp” and “REACTOME_PYROPTOSIS.v2023.1.Hs.grp” [82–84]. *MIR223* was removed from the ensuing analysis because it was not detected in the expression matrix of skin cutaneous melanoma (SKCM) cohort from TCGA (TCGA-SKCM, Table 1, colored gray). Thus, 74 genes remained for analysis. Genes derived from the literature can be functionally divided into inflammasome activation-related molecules, inflammasomes, adaptor proteins, cleavage proteins, executors, pore-forming-related proteins, released substances, and negative regulators (Table 1). Different forms of gene names, such as symbols and gene IDs, were also detailed in Table 1. The nonnegative matrix factorization (NMF) algorithm [85] was used to identify different expression patterns of PRGs and whether these genes have an impact on the survival rate of primary or metastatic melanoma patients. We calculated PScore based on significant genes screened by univariate Cox regression by using data for metastatic patients and by combining the expression correlations of these significant genes. Prognostic protective genes were selected due to clinical outcome and distinct expression patterns. Therefore, the PScore model that was established to represent the state of pyroptosis contained 31 genes: *AIM2*, *APIP*, *CASP1*, *CASP3*, *CASP4*, *CASP5*, *CASP8*, *CFLAR*, *CHMP2B*, *CHMP4A*, *CHMP5*, *DFNB59*, *GSDMB*, *GSDMD*, *GZMA*, *GZMB*, *IL18*, *IL1B*, *IRF1*, *IRF2*, *MEFV*, *NAIP*, *NLRC4*, *NLRP1*, *NLRP3*, *NLRP6*, *NLRP7*, *NOD2*, *TLR4*, *TNFRSF1B*, and *ZBP1*. Overall, PScore, which was built to computationally dissect the pyroptosis status of tissue samples and cell lines, was defined by the enrichment score of these genes calculated by ssGSEA using the R package “GSVA” (Additional file 2: Table S2) [84, 86]. PScore was validated by using 3 pyroptosis-related datasets from GEO.

**Table 1:** Pyroptosis-related genes used in this study

Symbol ID	Aliase or full name	Gene ID	Ensembl ID	Known functions reported by previous studies	Source	PMID
*NOD2*	*NLRC2*	64,127	ENSG00000167207	Activation of inflammasome	Literature	25,879,280
*TNFRSF1A*	*TNFR1*	7132	ENSG00000067182	Activation of inflammasome	Literature	25,879,280
*TNFRSF1B*	*TNFR2*	7133	ENSG00000028137	Activation of inflammasome	Literature	25,879,280
*TLR4*		7099	ENSG00000136869	Activation of inflammasome	Literature	25,879,280
*AIM2*		9447	ENSG00000163568	Inflammasome	Literature; MSigDB-GOBP_PYROPTOSIS	33,692,549
*NLRP1*		22,861	ENSG00000091592	Inflammasome	Literature; MSigDB-GOBP_PYROPTOSIS	33,692,549
*NLRP3*		114,548	ENSG00000162711	Inflammasome	Literature; MSigDB-GOBP_PYROPTOSIS	33,692,549
*ZBP1*		81,030	ENSG00000124256	Inflammasome	Literature; MSigDB-GOBP_PYROPTOSIS	32,729,116; 34,471,287
*NLRC4*		58,484	ENSG00000091106	Inflammasome	Literature; MSigDB-GOBP_PYROPTOSIS	33,692,549
*NAIP*	*BIRC1; NLRB1; psiNAIP*	4671	ENSG00000249437	Inflammasome	Literature; MSigDB-GOBP_PYROPTOSIS	31,662,274
*NLRP6*		171,389	ENSG00000174885	Inflammasome	Literature; MSigDB-GOBP_PYROPTOSIS	35,138,947
*NLRP7*		199,713	ENSG00000167634	Inflammasome	Literature	22,361,007
*NLRP9*		338,321	ENSG00000185792	Inflammasome	Literature; MSigDB-GOBP_PYROPTOSIS	35,138,947; 28,636,595; 28,731,031
*DHX9*		1660	ENSG00000135829	Inflammasome	Literature; MSigDB-GOBP_PYROPTOSIS	28,636,595; 28,731,031
*NLRP12*		91,662	ENSG00000142405	Inflammasome	Literature	32,838,963; 32,295,623
*MEFV*		4210	ENSG00000103313	Inflammasome	Literature; MSigDB-GOBP_PYROPTOSIS	25,879,280
*PYCARD*	*ASC*	29,108	ENSG00000103490	Adaptor protein	Literature; MSigDB-GOBP_PYROPTOSIS	33,692,549
*CASP1*		834	ENSG00000137752	Cleavage protein	Literature; MSigDB-GOBP_PYROPTOSIS; MSigDB-REACTOME_PYROPTOSIS	32,553,275; 32,109,412; 31,216,460
*CASP4*		837	ENSG00000196954	Cleavage protein	Literature; MSigDB-GOBP_PYROPTOSIS; MSigDB-REACTOME_PYROPTOSIS	32,109,412; 31,216,460; 22,895,188; 25,119,034
*CASP5*		838	ENSG00000137757	Cleavage protein	Literature; MSigDB-REACTOME_PYROPTOSIS	32,109,412; 31,216,460; 25,119,034
*CASP3*		836	ENSG00000164305	Cleavage protein	Literature; MSigDB-REACTOME_PYROPTOSIS	28,459,430; 32,188,940; 28,392,147
*CASP6*		839	ENSG00000138794	Cleavage protein	MSigDB-GOBP_PYROPTOSIS	
*CASP8*		841	ENSG00000064012	Cleavage protein	Literature; MSigDB-GOBP_PYROPTOSIS	30,381,458; 31,748,744; 31,723,262; 30,361,383; 34,012,073
*GZMA*		3001	ENSG00000145649	Cleavage protein	Literature; MSigDB-GOBP_PYROPTOSIS	32,299,851
*GZMB*		3002	ENSG00000100453	Cleavage protein	Literature; MSigDB-GOBP_PYROPTOSIS; MSigDB-REACTOME_PYROPTOSIS	32,188,940
*ELANE*		1991	ENSG00000197561; ENSG00000277571	Cleavage protein	Literature; MSigDB-GOBP_PYROPTOSIS; MSigDB-REACTOME_PYROPTOSIS	29,539,421
*CTSG*		1511	ENSG00000100448	Cleavage protein	Literature	33,692,549
*GSDMA*		284,110	ENSG00000167914	Executor	Literature; MSigDB-GOBP_PYROPTOSIS	29,362,479; 31,690,840; 29,695,864; 35,110,732
*GSDMB*		55,876	ENSG00000073605	Executor	Literature; MSigDB-GOBP_PYROPTOSIS	32,299,851; 29,362,479; 31,690,840
*GSDMC*		56,169	ENSG00000147697	Executor	Literature; MSigDB-GOBP_PYROPTOSIS	29,362,479
*GSDMD*		79,792	ENSG00000104518; ENSG00000278718	Executor	Literature; MSigDB-GOBP_PYROPTOSIS; MSigDB-REACTOME_PYROPTOSIS	32,553,275; 32,109,412; 26,375,003; 26,375,259; 26,611,636; 27,281,216; 27,383,986; 27,339,137; 27,418,190; 27,573,174; 29,195,811; 29,274,245; 29,362,479; 31,690,840
*DFNA5*	*GSDME*	1687	ENSG00000105928	Executor	Literature; MSigDB-GOBP_PYROPTOSIS; MSigDB-REACTOME_PYROPTOSIS	28,459,430; 32,188,940; 29,362,479; 31,690,840
*DFNB59*	*PJVK*	494,513	ENSG00000204311	Executor	Literature	29,362,479
*PLCG1*		5335	ENSG00000124181	Pore-forming	Literature	29,937,272
*RRAGA*	*RAGA*	10,670	ENSG00000155876	Pore-forming	Literature	34,289,345; 35,058,659
*RRAGC*	*RAGC*	64,121	ENSG00000116954	Pore-forming	Literature	34,289,345; 35,058,659
*LAMTOR1*	*C11orf59*	55,004	ENSG00000149357	Pore-forming	Literature	34,289,345; 35,058,659
*LAMTOR2*		28,956	ENSG00000116586	Pore-forming	Literature	34,289,345; 35,058,659
*MAPKSP1*	*LAMTOR3*	8649	ENSG00000109270	Pore-forming	Literature	34,289,345; 35,058,659
*C7orf59*	*LAMTOR4*	389,541	ENSG00000188186	Pore-forming	Literature	34,289,345; 35,058,659
*HBXIP*	*LAMTOR5*	10,542	ENSG00000134248	Pore-forming	Literature	34,289,345; 35,058,659
*FNIP2*		57,600	ENSG00000052795	Pore-forming	Literature	34,289,345; 35,058,659
*FLCN*	Folliculin	201,163	ENSG00000154803	Pore-forming	Literature	34,289,345; 35,058,659
*NINJ1*		4814	ENSG00000131669	Pore-forming	Literature	33,472,215
*IL1A*		3552	ENSG00000115008	Released substance	MSigDB-REACTOME_PYROPTOSIS	
*IL1B*		3553	ENSG00000125538	Released substance	Literature; MSigDB-REACTOME_PYROPTOSIS	29,195,811; 29,274,245
*IL18*		3606	ENSG00000150782	Released substance	Literature; MSigDB-REACTOME_PYROPTOSIS	29,195,811; 29,274,245
*HMGB1*		3146	ENSG00000189403	Released substance	Literature; MSigDB-REACTOME_PYROPTOSIS	33,692,549
*GPX4*		2879	ENSG00000167468	Negative regulators	Literature	29,937,272
*CHMP1A*		5119	ENSG00000131165	Negative regulators	Literature	30,467,171; 32,669,618
*CHMP1B*		57,132	ENSG00000255112	Negative regulators	Literature	30,467,171; 32,669,618
*CHMP2A*		27,243	ENSG00000130724	Negative regulators	Literature; MSigDB-REACTOME_PYROPTOSIS	30,467,171; 32,669,618
*CHMP2B*		25,978	ENSG00000083937	Negative regulators	Literature; MSigDB-REACTOME_PYROPTOSIS	30,467,171; 32,669,618
*VPS24*	*CHMP3*	51,652	ENSG00000115561	Negative regulators	Literature; MSigDB-REACTOME_PYROPTOSIS	30,467,171; 32,669,618
*CHMP4A*		29,082	ENSG00000254505; ENSG00000285302	Negative regulators	Literature; MSigDB-REACTOME_PYROPTOSIS	30,467,171; 32,669,618
*CHMP4B*		128,866	ENSG00000101421	Negative regulators	Literature; MSigDB-REACTOME_PYROPTOSIS	30,467,171; 32,669,618
*CHMP4C*		92,421	ENSG00000164695	Negative regulators	Literature; MSigDB-REACTOME_PYROPTOSIS	30,467,171; 32,669,618
*CHMP5*		51,510	ENSG00000086065	Negative regulators	Literature	30,467,171; 32,669,618
*CHMP6*		79,643	ENSG00000176108	Negative regulators	Literature; MSigDB-REACTOME_PYROPTOSIS	30,467,171; 32,669,618
*CHMP7*		91,782	ENSG00000147457	Negative regulators	Literature; MSigDB-REACTOME_PYROPTOSIS	30,467,171; 32,669,618
*KIAA0174*	*CHMP8; IST1*	9798	ENSG00000182149	Negative regulators	Literature	30,467,171; 32,669,618
*CFLAR*	*cFILP*	8837	ENSG00000003402	Negative regulators	Literature	32,193,329
*DDX3X*		1654	ENSG00000215301	Negative regulators	Literature	31,511,697
*MIR223*	MicroRNA 223	407,008	ENSG00000284567	Negative regulators	Literature; MSigDB-GOBP_PYROPTOSIS	23,772,809
*APIP*		51,074	ENSG00000149089		MSigDB-GOBP_PYROPTOSIS	
*ZAK*	*MAP3K20*	51,776	ENSG00000091436		MSigDB-GOBP_PYROPTOSIS	
*TREM2*		54,209	ENSG00000095970		MSigDB-GOBP_PYROPTOSIS	
*DPP9*		91,039	ENSG00000142002		MSigDB-GOBP_PYROPTOSIS	
*BAK1*		578	ENSG00000030110		MSigDB-REACTOME_PYROPTOSIS	
*TP63*		8626	ENSG00000073282		MSigDB-REACTOME_PYROPTOSIS	
*BAX*		581	ENSG00000087088		MSigDB-REACTOME_PYROPTOSIS	
*IRF1*		3659	ENSG00000125347		MSigDB-REACTOME_PYROPTOSIS	
*IRF2*		3660	ENSG00000168310		MSigDB-REACTOME_PYROPTOSIS	
*TP53*		7157	ENSG00000141510		MSigDB-REACTOME_PYROPTOSIS	
*CYCS*		54,205	ENSG00000172115		MSigDB-REACTOME_PYROPTOSIS	

1. MSigDB-GOBP_PYROPTOSIS: 28 genes

2. MSigDB-REACTOME_PYROPTOSIS: 27 genes

3. MSigDB-GOBP_PYROPTOSIS and MSigDB-REACTOME_PYROPTOSIS were downloaded from MSigDB, with the files named “MSigDB-GOBP_PYROPTOSIS.v2023.1.Hs.grp” and “MSigDB-REACTOME_PYROPTOSIS.v2023.1.Hs.grp”

$$\mathrm{PScore }=\mathrm{ ssGSEA}\_\mathrm{Score \,}(31\mathrm\,{ prognostic\,protective\,genes})$$

### Survival analysis and multivariate Cox regression analysis

The R package “survival” was used to perform survival analysis, with grouping based on NMF clusters, PScore groups, and/or mutation types. Considering the heterogeneity of the melanoma patients in different datasets, the “surv_cutpoint” function in the R package “survminer” was used to determine the optimal cutoff point and reduce the calculated batch effect. The parameter “minprop” refers to the minimal proportion of observations per group and was set as 0.4 and 0.2 when analyzing treatment-naïve and immune checkpoint blockade (ICB) therapy datasets, respectively. Then, age, sex, and stage were included as variables, and multivariate Cox regression model analysis was performed to assess whether PScore is an independent predictor in treatment-naïve cohorts. Kaplan‒Meier (KM) curves were used for survival analysis based on the log-rank test.

### Analysis of immune features and DNA damage features

The R package “MCPcounter” was used to evaluate immune infiltration reflected by microenvironment cell population (MCP) abundance [87]. The GEP score of each sample was computed based on the GEP gene signature from Ayers et al. [88] using GSVA. CYT scores were calculated by using the geometric mean of the gene expression of two cytolytic markers, *GZMA* and *PRF1* [89]. Stimulatory immune checkpoints, the richness of T/B-cell receptor (TCR/BCR) of TCGA-SKCM samples, aneuploidy, homologous recombination deficiency (HRD), and LOH_n_seg were acquired from Thorsson et al. [90] (https://gdc.cancer.gov/about-data/publications/panimmune); the files are “NIHMS958212-supplement-2.xlsx” and “NIHMS958212-supplement-7.xlsx.” Stimulatory immune checkpoints were obtained from column “Immune Checkpoint” and column “Gene” of “NIHMS958212-supplement-7.xlsx.” The others were from columns “Aneuploidy Score,” “Homologous Recombination Defects,” “BCR Richness” and “TCR Richness” of “NIHMS958212-supplement-2.xlsx.” LOH_n_seg is in the file “ABSOLUTE_scores.tsv.” For the cancer hallmark pathways, we obtained the gene set “h.all.v2023.1.Hs.symbols.gmt” from MSigDB [82–84] and performed pathway enrichment analysis using the fgsea package [91].

### Single-cell and spatial transcriptome analysis of melanoma datasets

The Seurat [92] and BayesSpace [32] packages were used to analyze single-cell transcriptome data and spatial transcriptome data, respectively. Spatial transcriptome data were preprocessed by performing PCA on the top 2000 most highly variable genes (HVGs) and then clustered based on the first seven principal components with 10,000 Markov chain Monte Carlo algorithm (MCMC) iterations. PScore was calculated using the PScore model algorithm described above.

### Statistical analyses

Pearson’s correlation *r* was used to measure statistical dependence between the normalized and log2-transformed expression levels of different genes. Correlation analyses between PScore and immune or DNA damage features were based on the Pearson method. The Wilcoxon rank sum test was employed to determine a significant difference in PScore between different groups. For multiple comparisons, the *p*-value was adjusted by using the BH method for multiple testing. Statistical analysis was performed using R (v4.1.2, https://cran.r-project.org/). The threshold for considering *p*-value or *p*-value corrected by the FDR method as significant was set at 0.05. For forest plot or Cox regression analysis, HR > 1 and *p*-value < = 0.05 indicated risk factors, and HR < 1 and *p*-value < = 0.05 indicated protective factors; *p*-value > 0.05 indicated nonsignificant factors. Specific details can be viewed in R codes (GitHub: https://github.com/Wenqiong9/melanoma_PScore).

## Results

### Pyroptosis can act as a prognostic factor in metastatic cutaneous melanoma

**Fig. 1:**
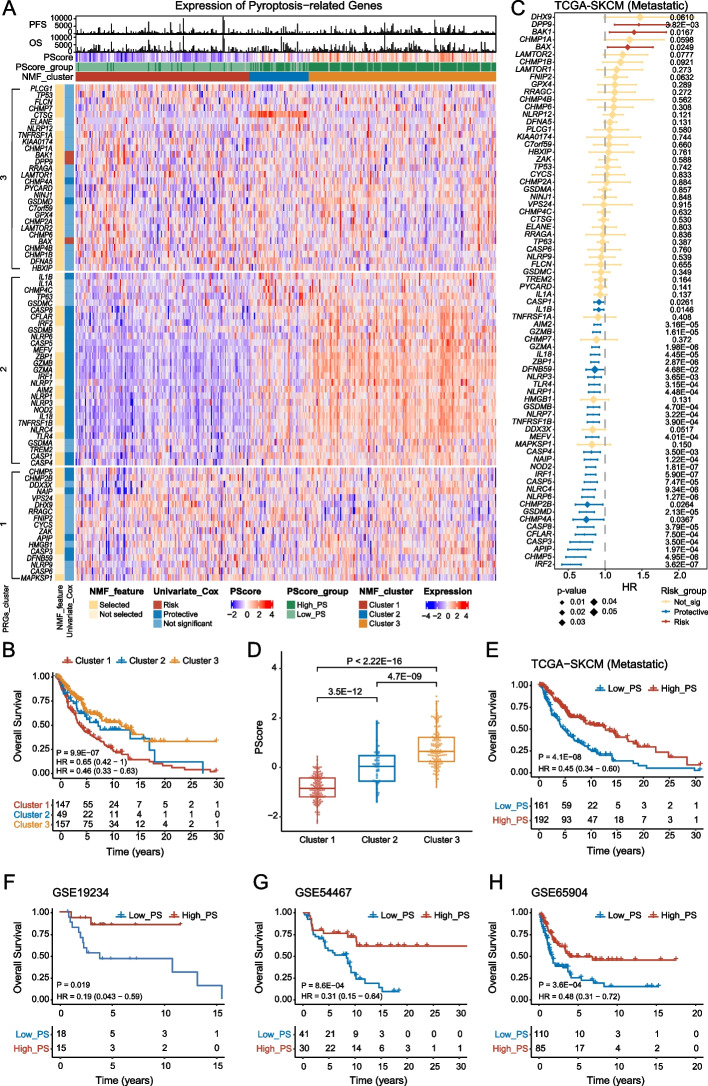
Clinical relevance of pyroptosis-related genes in metastatic cutaneous melanoma. **A** Heatmap showing expression of 74 PRGs in different NMF clusters or PScore groups of metastatic patients in the TCGA-SKCM cohort; red and blue denote high and low expression, respectively. The horizontal axis represents the patients, and the vertical axis represents the PRGs. Progression-free survival (PFS), overall survival (OS), PScore, and patient identities are shown above the heatmap, and the units of PFS and OS in the bar chart are days. Light green and green are used to represent “Low_PS” and “High_PS,” and the grouping threshold was determined by the surv_cutpoint function. Red, blue, and orange represent “Cluster 1,” “Cluster 2” and “Cluster 3” derived from NMF. The results of univariate Cox regression analysis of PRGs are annotated on the left, as well as the PRGs selected as features during NMF. PRGs were partitioned into three subclusters (PRGs_cluster) labeled 1, 2, and 3 on the vertical axis by k-means clustering. **B** Kaplan‒Meier (KM) curves for OS in metastatic melanoma patients stratified by the NMF algorithm (see the “Methods” section for statistical analysis). The figure above shows the KM curves, with the *x*-axis showing OS (unit: year) and the vertical axis showing OS rate. The colors of the KM curves represent different clusters. The figure below shows the number of patients at risk at the corresponding time point. **C** Forest plot showing univariate Cox regression analysis of 74 PRGs in metastatic melanoma patients. The *x*-axis represents HR, and the *y*-axis represents PRGs. Insignificant factors and significant protective and risk factors are shown in yellow, blue, and red, respectively. When HR > 1 and *p*-value < 0.05, the gene was considered a risk factor; when HR < 1 and *p*-value < 0.05, the gene was considered a protective factor. **D** Comparison of PScore across NMF-derived clusters. The *x*-axis represents NMF-derived clusters and the *y*-axis represents PScore. **E** KM curves for OS in metastatic melanoma patients stratified by PScore in TCGA-SKCM. **F**–**H** KM curves for OS in metastatic melanoma patients stratified by PScore using 3 independent validation datasets with traditional treatment

To explore the role of pyroptosis in melanoma patients, we collected 75 experimentally validated PRGs from the literature and MSigDB (Table 1) [4, 5, 9, 10, 18, 36, 37, 39–75, 82–84, 93]. By evaluating 74 PRGs (except for *MIR223*, see Methods), we found that these genes showed distinct expression patterns in primary and metastatic melanoma from the TCGA-SKCM cohort (Additional file 1: Fig. S1A, B). For both primary and metastatic melanoma, these genes were divided into three categories based on hierarchical clustering. Although the correlation was very high in metastatic disease, it was partially lost in primary disease. For example, *CASP4*, *NLRP1*, *MEFV*, *IL18*, and *NINJ1* correlated positively with *GSDMD* in metastatic melanoma patients but not in primary melanoma patients (Additional file 1: Fig. S1C). Thus, we performed NMF to classify primary and metastatic melanoma based on PRGs (Additional file 1: Fig. S1D, E). For metastatic melanoma, patients in cluster 3 with high expression of PRGs showed a survival advantage compared to those in cluster 1 (Fig. 1A, B, HR = 0.46, 95% CI = 0.33–0.63, log-rank test, *P* = 9.9E − 07). We further performed univariate Cox regression analysis for the 74 PRGs in melanoma and found that 34 PRGs had a significant effect on the OS of metastatic melanoma patients, with most acting as protective factors (Fig. 1C). Moreover, most of these PRGs showed significantly positive expression correlations, except for *BAK1*, *DPP9*, and *BAX*, which were unfavorable prognostic factors (Additional file 1: Fig. S1F). Many pairwise correlations between these 34 genes were not found in primary melanoma (Additional file 1: Fig. S1B, S1F), as mentioned above (Additional file 1: Fig. S1C). This suggests that these 31 PRGs, which are favorable prognostic factors, can serve as a signature gene set to indicate pyroptosis activity and predict prognosis. Thus, we constructed a pyroptosis-related gene score (PScore) model based on the 31 prognostic protective PRGs, most of which correlated positively based on ssGSEA [84, 86] (see the “Methods” section). We validated the performance and robustness of the PScore model by comparing it among known pyroptosis statuses in three publicly available datasets from GEO (GSE57253, GSE153494, and GSE192714). The results showed that samples under pyroptotic conditions had a significantly higher PScore than controls (Additional file 1: Fig. S2), indicating the robustness of the model. Cluster 2 and 3, with a favorable prognosis, had a significantly higher PScore than Cluster 1 (Fig. 1D; Wilcoxon rank-sum test, *P* < 2.2E − 16, *P* = 3.5E − 12), and metastatic melanoma patients with a higher PScore showed better OS (Fig. 1E, log-rank test, HR = 0.45, 95% CI = 0.34–0.60, *P* = 4.1E − 08). The reliability of PScore was validated in three independent melanoma cohorts obtained from GEO (Fig. 1F–H. GSE19234: HR = 0.19, 95% CI = 0.043–0.59, *P* = 0.019. GSE54467: HR = 0.31, 95% CI = 0.15–0.64, *P* = 8.6E − 04. GSE65904: HR = 0.48, 95% CI = 0.31–0.72, *P* = 3.6E − 04). To examine whether PScore can serve as an independent prognostic factor, we performed multivariate Cox regression analysis including it and clinical characteristics (e.g., age, sex, and tumor stage) and it to be a robust and independent prognostic biomarker for evaluating outcomes of metastatic melanoma (Additional file 1: Fig. S3A, HR = 0.39, 95% CI = 0.28–0.54, *P* < 0.001). This result was confirmed in three other publicly available independent cohorts (Additional file 1: Fig. S3B–D. GSE19234: HR = 0.14, 95% CI = 0.026–0.76, *P* = 0.023; GSE54467: HR = 0.46, 95% CI = 0.22–0.97, *P* = 0.041; GSE65904: HR = 0.42, 95% CI = 0.27–0.67, *P* < 0.001).

Primary melanoma was classified into 3 clusters based on expression levels of PRGs using NMF (Additional file 1: Figs. S1D, S4A), with no significant difference in survival among these clusters (Additional file 1: Fig. S4B). Additionally, three genes showed significant associations with OS (Additional file 1: Fig. S4C). Cluster 3 had high expression of these genes, such as *NOD2* and *IL18*, and high PScore (Additional file 1: Fig. S4D), but according to survival analysis, this model based on metastatic melanoma patients may not be suitable for primary melanoma patients (Additional file 1: Fig. S4E).

In summary, the results indicate that PScore can predict the prognosis of metastatic melanoma and be used to reflect the potential pyroptosis status based on transcriptome data.

### PScore is associated with druggable mutations

**Fig. 2:**
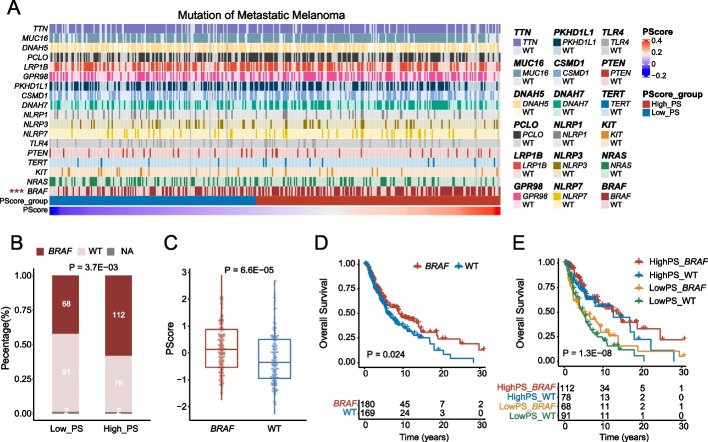
Combining clinical targetable mutations and PScore distinguishes the survival of *BRAF*-mutated melanoma patients. **A** Heatmap showing different mutation statuses and corresponding PScore groups of metastatic patients in the TCGA-SKCM cohort. The top 10 high-frequency mutations, common therapeutic targets, and PRGs with frequencies greater than 10% are displayed. Mutation marked with “***” on the left indicates significant differences in PScore. **B** Chi-square test for different PScore groups and *BRAF* mutations. The *x*-axis represents the PScore group, the *y*-axis represents the proportion of patients, and different colors in the bar graph represent *BRAF* mutation status. **C** Comparison of PScore between *BRAF*-mutated and non-*BRAF*-mutated groups in all metastatic melanoma. The *x*-axis represents *BRAF* mutation status, and the *y*-axis represents PScore. **D** Survival analysis based on whether *BRAF* is mutated in metastatic melanoma patients. The *x*-axis represents survival time (unit: year), the *y*-axis represents OS rate. **E** Survival analysis based on *BRAF* mutation and PScore groups in metastatic melanoma patients

CM has a high mutation load, and targeted therapy is an important therapeutic method for melanoma with driver gene alterations (i.e., *BRAF*, *NRAS*, and *KIT*). We next explored whether an interaction exists between pyroptosis and these clinically targetable driver mutations and high-frequency mutations (Additional file 1: Fig. S5A–B). First, the mutation status and PScore of the same patients were visualized using a heatmap, with a significantly higher *BRAF* mutation frequency in the group with a higher PScore (Fig. 2A, B). Next, we compared the PScore of the mutated and wild-type groups under different gene mutation conditions to determine any differences (Fig. 2C, Additional file 1: Fig. S5C, D). Melanoma patients with *BRAF* mutations also showed a significantly higher PScore than patients without *BRAF* mutations (Fig. 2C). The effect of *BRAF* mutation on metastatic melanoma is controversial [94, 95], though our study found that metastatic patients with *BRAF* mutations in the TCGA-SKCM cohort had favorable survival (Fig. 2D, *P* = 0.024). To further investigate the clinical implication of *BRAF* mutation and PScore, we divided patients into four groups and performed survival analysis by combining mutation status and PScore. The patients in the HighPS_*BRAF* group and HighPS_WT group had better prognosis (Fig. 2E, *P* = 1.3E − 08, Additional file 1: Fig. S5E, *P* < 0.001), suggesting that pyroptosis may be related to *BRAF* mutation and that promoting pyroptosis in patients and combining it with BRAF-targeted therapy may improve treatment outcomes.

### Pyroptosis is associated with cancer and immune features

**Fig. 3:**
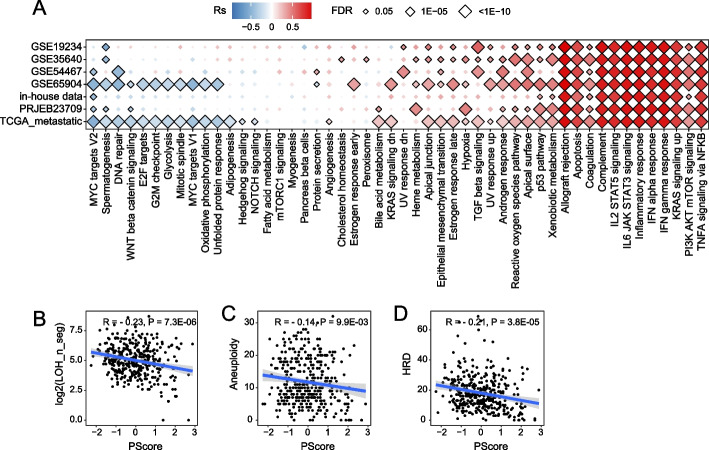
Pyroptosis is associated with cancer hallmark pathways and multiple cancer features. **A** Correlation of PScore with cancer hallmark pathways in multiple melanoma cohorts. The horizontal axis is the pathway names, and the vertical axis is the dataset identifiers. “TCGA_metastatic” refers to metastatic TCGA-SKCM patients. Red and blue denote high and low correlation coefficients, respectively. Significant points are labeled using black diamonds. **B**-**D** Correlation of PScore with DNA damage measures in metastatic melanoma. The *x*-axis is PScore, and the *y*-axis represents loss of heterozygosity (LOH), aneuploidy, and homologous recombination deficiency (HRD) from left to right. Each point represents a patient, and the blue line is the fitted correlation line. Log10(LOH_n_seg) denotes the LOH data were log10-transformed

To explore the mechanism of pyroptosis in melanoma, we performed association analysis between PScore and the enrichment score of hallmark gene sets from MSigDB [82–84] across seven melanoma datasets. We observed significantly positive correlations between PScore and immune-related hallmark pathways in all datasets, including TNFA_signaling_via_NFKB, KRAS_signaling_up, IFN_gamma_response, IFN_alpha_response, inflammatory_response, IL6_JAK_STAT_signaling, IL2_STAT5_signaling, and complement pathway, among others (Fig. 3A). In contrast, multiple carcinogenic signaling pathways were found to correlate significantly negatively with PScore in multiple datasets, such as the MYC_targets_V2 and DNA_repair pathways (Fig. 3A). In addition, PScore showed a slightly negative correlation with chromatin instability (Fig. 3B–D), including loss of heterozygosity (LOH), aneuploidy, and homologous recombination deficiency (HRD).

**Fig. 4:**
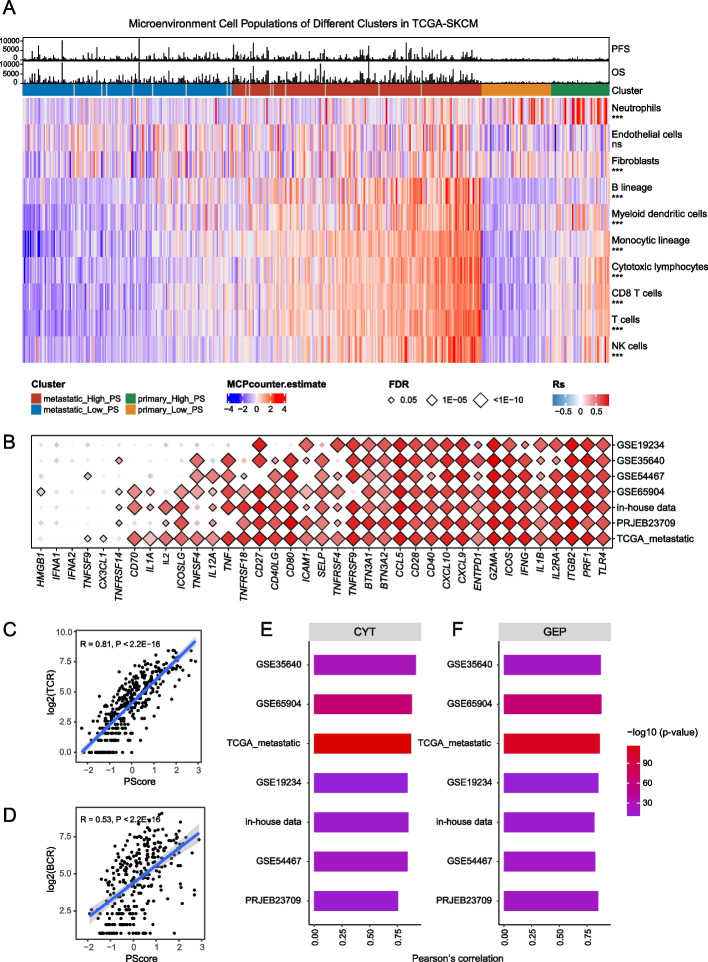
Pyroptosis is associated with multiple immune features. **A** Heatmap showing the microenvironmental cell population of different clusters in TCGA-SKCM patients. The *x*-axis represents different patients, and these patients were divided into 4 clusters. In each cluster, patients were ranked by PScore from lowest to highest. Cell types are annotated on the right of the plot. *** indicates *P* < 0.001 and represents a significant difference in the degree of immune infiltration between the “metastatic_High_PS” and “metastatic_Low_PS” groups. Red and blue denote high and low expression, respectively. Progression-free survival (PFS), overall survival (OS), and patient clusters are shown above the heatmap. **B** Correlation of PScore with stimulated immune checkpoints in multiple melanoma cohorts. Red and blue indicate the magnitudes of correlations: red, high; blue, low. Significant points are labeled using black diamonds. The horizontal axis is the checkpoints, and the vertical axis is the dataset identifiers. “TCGA_metastatic” refers to metastatic TCGA-SKCM patients. (C-D) Correlation of PScore with TCR/BCR diversity in metastatic TCGA-SKCM patients. The *x*-axis is PScore, and the *y*-axis is log2(TCR/BCR diversity). Each point represents a patient, and the blue line is the fitted correlation line. **E**–**F** Bar plots showing the correlation between PScore and cell toxicity signatures (**E**: CYT; **F**: GEP) in metastatic TCGA-SKCM and GEO datasets. The *x*-axis represents the Pearson correlation coefficient, and the *y*-axis represents different datasets; the color of the bar graph represents the − log10 (*p*-value)

Regarding immune features, PScore was also associated with immune cell infiltration, as calculated using the MCP-counter method. NK cells, T cells, cytotoxic lymphocytes, monocytic lineage cells, and B lineage cells were enriched in the metastatic_High_PS group compared with the metastatic_Low_PS group (Fig. 4A). The correlation between PScore and these cell types was validated across seven datasets (Additional file 1: Fig. S6) showing that pyroptosis correlated positively with most stimulatory immune checkpoints (Fig. 4B), such as *CXCL9* and *ICOS*, which are associated with T-cell activation, and *IFNG*, which plays crucial roles in activating effector immune cells and enhancing antigen presentation [96]. PScore was also positively related to T-cell receptor richness (TCR, Fig. 4C), B-cell receptor richness (BCR, Fig. 4D), cytolytic activity (CYT, Fig. 4E), and the T-cell-inflamed gene expression profile (GEP, Fig. 4F). These results indicate that pyroptosis may be closely related to immunogenicity and that patients with a high PScore may tend to have “hot tumors,” accompanied by higher TCR/BCR clone richness, CYT score, GEP level, and thus an activated immune microenvironment. These features may explain, at least in part, the survival advantage of melanoma patients with a higher PScore.

### Heterogeneity of pyroptosis status in the TME at the bulk/single-cell and spatial transcriptome levels

**Fig. 5:**
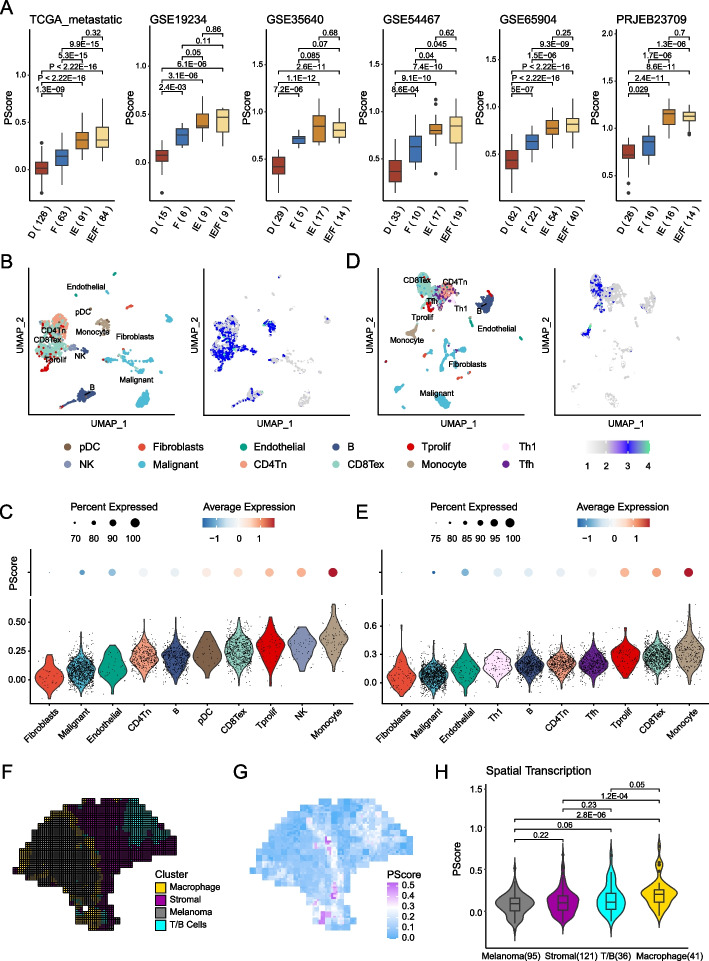
Single-cell and spatial transcriptomic landscapes of PScore. **A** Boxplots show PScore in different TME subtypes of TCGA and five other datasets, including GSE19234, GSE35640, GSE54467, GSE65904, and PRJEB23709. The *x*-axis is TME subtype, and the *y*-axis is PScore. **B**, **D** Dimensionality reduction plots and feature plots of PScore in two melanoma single-cell datasets. The same cell types in both datasets are colored with the same color. The change in the gray, blue, and cyan colors in the legend represents the change in PScore from low to high. **C**, **E** Violin plots and dot plots to visualize PScore in different cell types. The *x*-axis is the TME subtype, and the *y*-axis of the violin plot is PScore. The color of different cells is the same as the dimensionality reduction plots above. For dot plot, the changes in the blue, white, and red colors in the legend represent the change in PScore from low to high, and the size of the dot indicates the expression ratio. **F**, **G** Cell type annotation and corresponding level of PScore in spatial transcriptome data. The whole tissue plane is annotated into four cell types: macrophages (yellow), stromal cells (purple), melanoma cells (dark gray), and T/B cells (cyan). Blue, white, and purple indicate the magnitudes of PScore: blue, low; purple, high. **H** Violin plot to visualize comparison of PScore in different clusters consistent with (**F**). The *x*-axis is cell type, and the *y*-axis is PScore

As we found pyroptosis to be associated with multiple immune response pathways and immune cells (Figs. 3A and 4A), we further dissected the relationships and assessed the four TME subtypes [97] that act as generalized immunotherapy biomarkers across many cancer types (“immune-depleted” (D)-; “fibrotic” (F)-; “immune-enriched, nonfibrotic” (IE)-; and “immune-enriched, fibrotic” (IE/F)-TME subtypes) in multiple independent melanoma datasets (Fig. 5A). We observed that PScore in the D-TME subtype was consistently lower than that in the other three subtypes across the six melanoma datasets. This result suggests that low-PScore tumors may represent “cold tumors,” which are associated with immunotherapy resistance.

Considering the heterogeneity of the TME, we further explored pyroptosis status at a single-cell resolution. We built PScore to represent the pyroptosis status for each single cell according to the same method as described above; for GSE115878, a variety of cells underwent pyroptosis with obviously different PScore (Fig. 5B–C). PScore was generally lowest for malignant cells and fibroblasts but higher for immune cells, such as T cells, monocytes, and B cells. Consistent results were observed when using another independent melanoma single-cell RNA-seq dataset (Fig. 5D–E, GSE72056).

Although scRNA-seq achieves high-throughput and single-cell level profiling of gene expression, spatial information is not retained because tissue is dissociated for sample preparation. To investigate the spatial characteristics of pyroptosis in the TME, we obtained spatial transcriptome melanoma data and clustered and annotated cell types, as previously described [32]. We observed that annotated macrophages (yellow) and a few T/B cells (cyan) infiltrated around the tumor cells (gray, Fig. 5F). Interestingly, PScore was higher in the tumor border with high infiltration of macrophages and in regions enriched with T/B cells than in the tumor center and stromal regions (Fig. 5G, H). Moreover, the expression of key genes for pyroptosis, such as *AIM2*, *GSDMD*, *IL18*, *IRF1*, and *NLRP1*, exhibited trends similar to PScore (Additional file 1: Figs. S7, S8). Furthermore, different cells were dominated by different pyroptotic pathways. Regarding caspases, tumor cells exhibited predominantly high expression of *CASP4*, and immune cells showed high expression of *CASP1*, *CASP4*, and *CASP8*, while *CASP5* plays an important role in stromal cells (Additional file 1: Fig. S8). All of these findings indicate heterogeneity of pyroptosis status among TME cell populations; in particular, there was a predominant difference in PScore between malignant cells and immune cells. Thus, inducing pyroptosis in tumor cells may be a new cancer treatment strategy.

### Correlation between pyroptosis status and immunotherapy efficacy

**Fig. 6:**
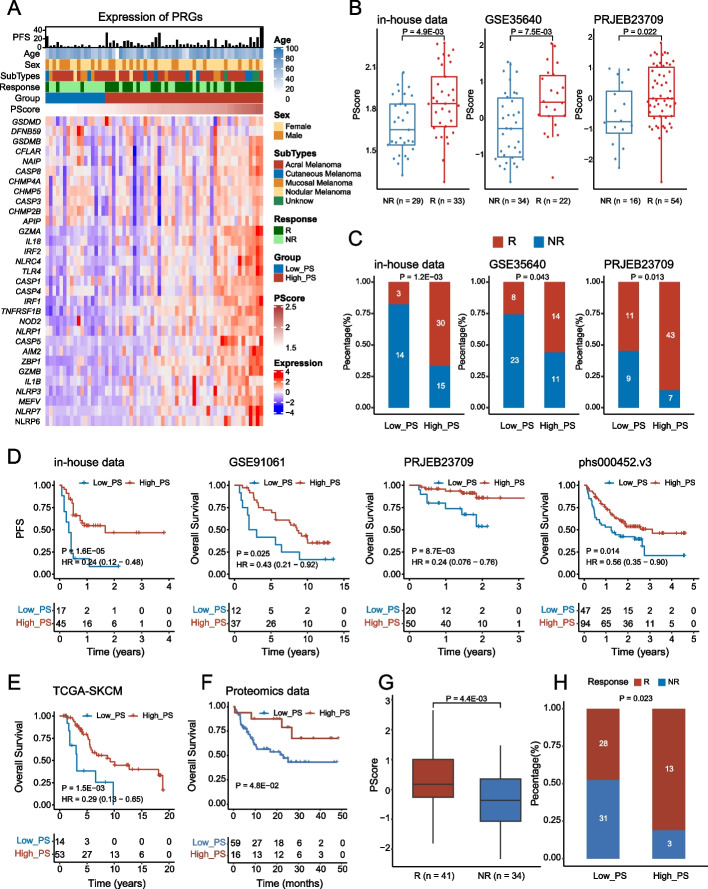
Clinical relevance of PScore in melanoma cohorts treated with ICB therapy. **A** Heatmap showing expression of 31 PRGs and corresponding clinical features of our in-house data; red and blue denote high and low expression, respectively. The horizontal axis represents the patients, and the *y*-axis represents the PRGs. Legends on the right, including age, sex, subtype, immune response, PScore, and PScore group, are annotated above the heatmap. **B** Boxplots show PScore in different immune responses of in-house data, PRJEB23709 and GSE35640. The *x*-axis represents the immune response (NR, nonresponder; R, responder). **C** Bar plots show chi-square test results of immune response and PScore groups of in-house data, PRJEB23709 and GSE35640. The *x*-axis represents the PScore group, the *y*-axis represents the proportion of patients, and the colors represent the immune response. **D**–**F** KM curves for PFS or OS of in-house data, GSE91061, PRJEB23709, phs000452.v3, TCGA-SKCM patients with immunotherapy and proteomics data. The *x*-axis represents survival time (unit: year or month), and the *y*-axis represents OS or PFS rate. **G** Boxplots show PScore in different immunotherapy responses of proteomics data. The *x*-axis represents the immune response, and the *y*-axis represents PScore. **H** Bar plot showing the chi-square test results of immunotherapy response and PScore groups of proteomics data. The *x*-axis represents the PScore group, and the *y*-axis represents the proportion of patients

To verify whether PScore can predict the clinical outcomes of melanoma patients treated with immunotherapy, we visualized expression of the PRGs used to calculate PScore in our in-house data cohort [31] and observed higher expression in patients who responded to anti-PD1 immunotherapy than in nonresponders (Fig. 6A). Then, we found that immunotherapy responders had a significantly higher PScore than nonresponders in our in-house data and two other independent ICB treatment datasets [98, 99] (Fig. 6B, in-house data: *P* = 4.9E − 03; GSE35640: *P* = 7.5E − 03; PRJEB23709: *P* = 0.022). In addition, a significantly higher percentage of responders was found in the High_PS group in these three ICB treatment datasets (Fig. 6C, in-house data: *P* = 1.2E − 03; GSE35640: *P* = 0.043; PRJEB23709: *P* = 0.013). Survival analyses showed that patients with a high PScore had significantly better therapeutic outcomes than those with a low PScore, consistent with the previous results (Fig. 6D–E, in-house data: HR = 0.24, 95% CI = 0.12–0.48, *P* = 1.6E − 05; GSE91061: HR = 0.43, 95% CI = 0.21–0.92, *P* = 0.025; PRJEB23709: HR = 0.24, 95% CI = 0.076–0.76, *P* = 8.7E − 03; phs00452.v3: HR = 0.56, 95% CI = 0.35–0.90, *P* = 0.014; TCGA: HR = 0.29, 95% CI = 0.13–0.65, *P* = 1.5E − 03). Finally, we performed the analysis using protein data and obtained the same results (Fig. 6F–H, 6F: *P* = 0.048; 6G: *P* = 4.4E − 03; 6H: *P* = 0.023). These findings suggest that pyroptosis is a protective factor in melanoma and has the potential to serve as a marker for immunotherapy response.

## Discussion

Pyroptosis has received extensive attention because of its great potential in cancer treatment [14], and there are at least 6 relevant clinical trials in progress [100]. In this study, we constructed the PScore model to evaluate pyroptosis status in melanoma patients, as there is as yet no applicable and quick method to estimate pyroptosis status other than electron microscopy or PCR [101–103]. Although some pyroptosis-related models based on RNA sequencing [104, 105] have been explored to predict the clinical outcome of cutaneous melanoma patients, in-depth research is still lacking. We found that PRGs have different expression patterns in primary and metastatic tumors and that the NMF method based on PRGs can predict the prognosis of metastatic patients but not primary patients. By integrating the single-cell and spatial transcriptome, we revealed the heterogeneity of the expression patterns of pyroptosis-related genes at single-cell and spatial levels, partially explaining the side effects of pyroptosis drugs. We analyzed the performance of this model in metastatic melanoma patients receiving immunotherapy and found that it can serve as a predictive model for the immune response in melanoma patients treated with immunotherapy, including one in-house and three public RNA-seq cohorts and one proteomics cohort.

First, we collected PRGs as comprehensively as possible based on previous studies (Table 1). Compared with published pyroptosis-related prognostic models, PScore showed superior performance in multiple independent datasets (Additional file 1: Fig. S9A, B, Additional file 2: Table S3). We also investigated whether a larger initial gene set leads to better model performance. We analyzed the correlation between the number of input genes and the c-index and found no significant correlation (Additional file 1: Fig. S10).

Moreover, considering the difference between primary and metastatic tumors, we analyzed them separately and found that PRGs indeed have different manifestations in primary and metastatic melanoma. Most PRGs act as protective factors in metastatic melanoma but not significantly in primary melanoma. BRAF kinase is the core component of the RAS-RAF-MEK-ERK signaling cascade (MAPK signaling) pathway, and *BRAF* mutation generally indicates poor prognosis. We conclude that PScore is related to *BRAF* mutation and KRAS signaling, and we infer that pyroptosis protects against overactivated MAPK signaling. A previous study also supports the protective effect of pyroptosis on melanoma: BRAFi and MEKi targeted therapy induces GSDME cleavage and mediates pyroptosis, but BRAFi + MEKi-resistant disease lacks pyroptosis markers [14], suggesting possible interaction with pyroptosis and the RAS-RAF-MEK-ERK (MAPK) pathway. However, whether pyroptosis is directly involved in MAPK pathway regulation or has an indirect correlation needs further study.

Furthermore, studies on pyroptosis are focusing on inducing tumor cell pyroptosis by GSDME cleavage [106, 107]. Our single-cell analyses suggest that pyroptosis can occur in any type of cell and that inducing “repairable” pyroptosis in immune cells may provide a new perspective for future treatment. Our results suggest an association between pyroptosis and immunotherapy response, indicating the potential of pyroptosis in tumor treatment. We investigated the heterogeneity of pyroptosis levels among different components of the TME and observed significantly distinct levels, whereby tumor cells showed a significantly lower PScore than immune cells and immune cell subpopulations displayed different levels of pyroptosis. All of the above findings indicate that the whole TME, rather than a specific cell type such as monocytes, affects the prognosis of patients. Nonetheless, it is still meaningful to study the role of pyroptotic tumor-associated macrophages (TAMs) in tumor progression because the TAM PScore was the highest, which makes these cells relatively easy to study. Spatial transcriptome data also revealed a consistent pattern of pyroptosis in the TME. The pyroptosis status of immune cells was higher than that of tumor cells, also suggesting potential side effects when using pyroptosis-inducing drugs. Ideally, we should only induce pyroptosis of bad players (tumor cells and immune or stromal cells that promote tumor growth), whereas immune cells or stromal components that have antitumor effects should be protected from pyroptosis or be repaired. We look forward to future development of pyroptosis-targeting drugs, and we anticipate that nanoparticles and aptamers loaded with pyroptosis-related drugs that can be selectively enriched in tumor cells will be available in the future.

### Limitations

The mechanism of pyroptosis primarily involves the activation of protein cleavage. Our model predominantly relies on transcriptome data. Although we did endeavor to incorporate proteome data, importantly, only a subset of model features could be identified using the protein dataset. As a result, additional validation at the protein level remains imperative for verification.

## Conclusions

A variety of cells undergoing pyroptosis in melanoma constitute a heterogeneous environment for tumors. Pyroptosis can act as a predictor of survival and immune response and a therapeutic target in melanoma patients.

### Supplementary Information

**Additional file 1:** **Table S1.** Summary of human melanoma datasets. **Fig. S1.** Expression pattern and NMF clustering of PRGs in TCGA-SKCM. (A) Gene expression correlation of 74 pyroptosis-related genes in primary (bottom left) and metastatic (top right) samples. Blue and red indicate the magnitudes of correlations: blue, high; red, low. Significant points are labeled using *. Hierarchical clustering based on gene expression was used. (B) The bar graph shows the number of PRGs correlating positively or negatively with each PRG, with the primary tumor group on the left and metastatic tumor group on the right. The X axis represents the number of positively or negatively correlating PRGs, and the Y axis represents the PRGs. The positive and negative signs of the X axis coordinates represent positive or negative correlations. A significant correlation was considered when the absolute value of Pearson correlation coefficient was greater than 0.3 and *p*-value < 0.05. (C) Venn diagram showing PRGs correlating with GSDMD expression in primary and metastatic tumors. The number of genes in each part of the Venn diagram and their percentage in the overall population (26 genes) are indicated in the figure. Metastatic: 13 (50%); overlap: 9 (34.6%); primary: 4 (15.4%). (D-E) Optimal NMF clustering of mRNA expression profiles for primary (D) and metastatic (E) samples. The left image shows the parameters of NMF clustering, and the optimal rank was selected as the front point of the line segment with the minimum slope in the cophenetic plot. The image on the right is the consensus matrix drawn based on the features extracted by the NMF algorithm. (F) Gene expression correlation of 34 PRGs in metastatic samples. Both the horizontal and vertical axes are 34 PRGs. Blue and red indicate the magnitudes of the correlations: blue, high; red, low. Significant points are labeled using black asterisks. **Fig. S2.** Validation of the PScore model using 3 pyroptosis-related datasets. Pyroptosis-related datasets, including GSE57253, GSE153494 and GSE192714, were obtained from GEO, and PScore was calculated with significant genes using univariate Cox regression analysis. The x axis represents the control group and experimental groups in the corresponding dataset. PScore was significantly higher in the experimental groups than the control group (GSE57253: left, Kruskal‒Wallis, *P* = 5.7E-03; GSE153494: middle, Kruskal‒Wallis, *P* = 0.014; GSE192714: right, Wilcoxon, *P* = 0.029). In GSE57253, NLRC4-macrophage activation syndrome (MAS) and neonatal-onset multisystem inflammatory disease (NOMID) are related to NLRC4 or NLRP3, respectively, and accompanied by IL-1β and IL-18 overproduction and increased pyroptosis. GSE153494 was used to describe the progression status of myocardial infarction associated with pyroptosis over time. Mll4 knockout elicits GSDMD-mediated pyroptosis in GSE192714. **Fig. S3.** Forest plot representation of multivariate Cox regression analyses using 4 datasets. Multivariate Cox regression analyses of PScore and 3 other variables, including sex, age and stage, in TCGA-SKCM (A) and 3 independent melanoma datasets, including GSE19234 (B), GSE54467 (C) and GSE65904 (D). Risk factors: HR > 1 and *p*-value <= 0.05; protective factors: HR < 1 and *p*-value <= 0.05; nonsignificant factors: *p*-value > 0.05. **Fig. S4.** Clinical relevance of PRGs in primary cutaneous melanoma. (A) Heatmap showing expression of 74 PRGs in different NMF clusters or PScore groups of primary patients in the TCGA-SKCM cohort; red and blue denote high and low expression, respectively. The horizontal axis represents the individual patients, and the vertical axis represents the PRGs. PFS, OS and patient identities are shown above the heatmap. Light green and green are used to represent the ‘Low_PS’ and ‘High_PS’ groups. Red, blue and orange represent ‘Cluster_1’, ‘Cluster_2’ and ‘Cluster_3’ derived from NMF. The results of univariate Cox regression analysis of PRGs are annotated on the left, as are the PRGs selected as features during NMF. PRGs were divided into three subclusters (PRGs_cluster) using k-means clustering. (B) KM curves for OS in primary samples stratified by the NMF algorithm. The x-axis represents survival time (unit: year), and the y-axis represents OS rate. The colors of the KM curves represent different NMF-derived clusters. (C) Forest plot showing univariate Cox regression analysis of OS with 74 PRGs in primary melanoma patients. The x-axis represents the HR, and the y-axis represents the different PRGs. Yellow and blue indicate nonsignificant and protective genes, respectively. Protective genes: HR < 1 and *p*-value <= 0.05; nonsignificant genes:* p*value > 0.05. (D) Comparison of PScore across NMF-derived clusters. The x-axis represents NMF-derived clusters, and the y-axis represents PScore. Colors correspond to the heatmap (A) and KM curve (B). (E) Kaplan‒Meier curves for OS in primary samples stratified by PScore. The x-axis represents survival time (unit: year), and the y-axis represents OS rate. The colors of the KM curves represent different PScore groups. **Fig. S5.** Combining druggable mutations and PScore distinguishes the survival of metastatic *BRAF*-mutated melanoma patients. (A-B) Mutation landscapes of the top 10 high-frequency mutations and PRGs. The horizontal axis of the heatmap represents the patients, and the vertical axis represents the genes. (C-D) Boxplots show PScore in different mutation statuses in metastatic TCGA-SKCM. C: Top 10 high-frequency genes. D: Common therapeutic targets and top four PRGs. The x-axis represents mutation statuses, and the y-axis represents PScore. (E) Forest plot showing the HR of survival analysis related to Figure 2E. Risk factors: HR > 1 and *p*-value <= 0.05; protective factors: HR < 1 and *p*-value <= 0.05; nonsignificant factors: *p*value > 0.05. **Fig. S6.** Immune cell scores calculated with MCPcounter for seven datasets. Correlation of PScore with immune cell scores in multiple melanoma cohorts. The x-axis represents the type of infiltrating cells, and the y-axis represents the different datasets. ‘TCGA_metastatic’ refers to metastatic TCGA-SKCM patients. The color of the lattice represents the correlation coefficient. Significant points are labeled using black diamonds. **Fig. S7.** Violin plots showing expression of the 31 genes used to calculate PScore corresponding to Figure 5B-E. (A) GSE115978, (B) GSE72056. The x-axis represents the 31 PRGs, and the y-axis represents the different cell subpopulations. The colors of the violin plot reflect cell types that are consistent with Figure 5B-E. **Fig. S8.** Spatial expression of the 31 genes used to calculate PScore corresponding to Figure 5G-H. Blue, white and purple indicate the magnitudes of PRGs: blue, low; white, middle; purple, high. The corresponding cell annotations can be found in Figure 5F. These genes are sorted alphabetically. **Fig. S9.** Comparisons of the PScore model and other pyroptosis-related models. (A) Forest plot showing HR and confidence intervals of different methods in multiple datasets. The horizontal axis represents log10(HR), and the vertical axis represents different methods and datasets. (B) Bar chart showing the c-index of different methods in multiple datasets. The c-index performance of 11 methods, including ssGSEA using 31 protective PRGs (PScore) and 10 obtainable pyroptosis-related models, were compared across four datasets. The horizontal axis represents different methods, the vertical axis represents the c-index, and the colors of the bar graph represent different datasets. **Fig. S10.** Correlation between the number of input genes and the c-index. The correlation of gene input size for the 11 methods mentioned in Fig. S9 with concordance (c-index) across different datasets was analyzed. The x-axis represents the number of genes used in each method, and the y-axis represents the c-index.

**Additional file 2:** **Table S2.** PScore of all samples in different datasets. Datasets: in-house data, TCGA_metastatic, GSE19234, GSE35640, GSE54467, GSE65904, GSE115978, GSE72056, PRJEB23709, 2018_thrane_melanoma. Note: PScore was calculated for all samples in the single-cell datasets (GSE115978, GSE72056), but only metastatic samples were used in subsequent analyses. **Table S3.** HR and c-index of pyroptosis-related models.

## Data Availability

All data used in our analyses were described in the “Meth” section in the “collection and processing” section. The resources and tools used in our analyses were described in each method section in the methods.

## Abbreviations

BCR: The B cell receptor richness
CM: Cutaneous melanoma
CYT: Cytolytic activity
dbGaP: The database of genotypes and phenotypes
D-TME: Immune-depleted tumor microenvironment subtypes
F-TME: Fibrotic TME subtypes
GEO: Gene-expression omnibus
GEP: The T cell-inflamed gene expression profile
GSDM: Gasdermin
HR: Hazard ratio
HRD: Homologous recombination deficiency
HVGs: Highly variable genes
ICB: Immune checkpoint blockade
IE/F-TME: Immune-enriched, fibrotic TME subtypes
IE-TME: Immune-enriched, nonfibrotic TME subtypes
KM curve: Kaplan–Meier curve
Lasso: Least absolute shrinkage and selection operator
LOH: Loss of heterozygosity
MCMC: Markov chain Monte Carlo algorithm
MCP: Microenvironment cell populations
MDSCs: Myeloid-derived suppressor cells
MSigDB: The molecular signatures database
NK: Natural killer cell
NMF: Nonnegative matrix factorization
OS: Overall survival
PFS: Progression-free survival
PRGs: Pyroptosis-related genes
PScore: Pyroptosis-related gene score
SKCM: Skin cutaneous melanoma
SRA: Sequence read archive
ssGSEA: Single-sample gene set enrichment analysis
TAM: Tumor-associated macrophage
TCGA: The cancer genome atlas
TCR: The richness of T cell receptor
TMAO: Trimethylamine N-oxide
TME: Tumor immune microenvironment
