# Supplementary material for: Multiomics characterization of pyroptosis in the tumor microenvironment and therapeutic relevance in metastatic melanoma
Source: BMC Med. 2024 Jan 17;22:24. doi: 10.1186/s12916-023-03175-0 (PMC10792919; doi:10.1186/s12916-023-03175-0)
Supplement: Supplementary file 1 — Additional file 1: Table S1. Summary of human melanoma datasets. Fig. S1. Expression pattern and NMF clustering of PRGs in TCGA-SKCM. (A) Gene expression correlation of 74 pyroptosis-related genes in primary (bottom left) and metastatic (top right) samples. Blue and red indicate the magnitudes of correlations: blue, high; red, low. Significant points are labeled using *. Hierarchical clustering based on gene expression was used. (B) The bar graph shows the number of PRGs correlating positively or negatively with each PRG, with the primary tumor group on the left and metastatic tumor group on the right. The X axis represents the number of positively or negatively correlating PRGs, and the Y axis represents the PRGs. The positive and negative signs of the X axis coordinates represent positive or negative correlations. A significant correlation was considered when the absolute value of Pearson correlation coefficient was greater than 0.3 and p-value < 0.05. (C) Venn diagram showing PRGs correlating with GSDMD expression in primary and metastatic tumors. The number of genes in each part of the Venn diagram and their percentage in the overall population (26 genes) are indicated in the figure. Metastatic: 13 (50%); overlap: 9 (34.6%); primary: 4 (15.4%). (D-E) Optimal NMF clustering of mRNA expression profiles for primary (D) and metastatic (E) samples. The left image shows the parameters of NMF clustering, and the optimal rank was selected as the front point of the line segment with the minimum slope in the cophenetic plot. The image on the right is the consensus matrix drawn based on the features extracted by the NMF algorithm. (F) Gene expression correlation of 34 PRGs in metastatic samples. Both the horizontal and vertical axes are 34 PRGs. Blue and red indicate the magnitudes of the correlations: blue, high; red, low. Significant points are labeled using black asterisks. Fig. S2. Validation of the PScore model using 3 pyroptosis-related datasets. Pyroptosis-related d [file 12916_2023_3175_MOESM1_ESM.pdf]

Table S1. Summary of human melanoma datasets

	TCGA-SKCM (N = 469)	GSE19234 (N = 39)	GSE54467 (N = 79)	GSE65904 (N = 198)	in-house data (N = 62)	GSE35640 (N = 65)	PRJEB23709 (N = 70)	GSE91061 (N =49)	phs000452.v3 (N = 141)	GSE72056 (N = 19)	GSE115978 (N = 11)	2018_thrane_melanoma (N = 1)	Proteomics data (N = 185)	Overall (N = 1388)
**gender**														
Male	289 (61.6%)	0 (0%)	50 (63.3%)	116 (58.6%)	23 (37.1%)	0 (0%)	44 (62.9%)	0 (0%)	0 (0%)	12 (63.2%)	6 (54.5%)	1 (100%)	108 (58.4%)	649 (46.8%)
Female	180 (38.4%)	0 (0%)	29 (36.7%)	81 (40.9%)	39 (62.9%)	0 (0%)	26 (37.1%)	0 (0%)	0 (0%)	7 (36.8%)	5 (45.5%)	0 (0%)	77 (41.6%)	444 (32.0%)
Missing	0 (0%)	39 (100%)	0 (0%)	1 (0.5%)	0 (0%)	65 (100%)	0 (0%)	49 (100%)	141 (100%)	0 (0%)	0 (0%)	0 (0%)	0 (0%)	295 (21.3%)
**age**														
Mean (SD)	58.2 (15.7)	63.1 (18.1)	56.2 (15.2)	61.5 (14.3)	58.9 (13.2)	NA (NA)	57.5 (12.6)	NA (NA)	NA (NA)	68.1 (11.4)	66.4 (16.3)	NA (NA)	57.0 (15.5)	58.8 (15.3)
Median [Min, Max]	58.0 [15.0, 90.0]	65.0 [30.0, 92.0]	57.0 [19.0, 84.0]	63.0 [22.0, 91.0]	61.0 [11.0, 85.0]	NA [NA, NA]	57.0 [24.0, 81.0]	NA [NA, NA]	NA [NA, NA]	67.0 [43.0, 86.0]	73.0 [37.0, 86.0]	NA [NA, NA]	58.3 [20.0, 89.2]	59.3 [11.0, 92.0]
Missing	8 (1.7%)	0 (0%)	0 (0%)	3 (1.5%)	0 (0%)	65 (100%)	0 (0%)	49 (100%)	141 (100%)	0 (0%)	0 (0%)	1 (100%)	0 (0%)	267 (19.2%)
**stage (AJCC)**														
Stage 0	7 (1.5%)	0 (0%)	0 (0%)	0 (0%)	0 (0%)	0 (0%)	0 (0%)	0 (0%)	0 (0%)	0 (0%)	0 (0%)	0 (0%)	0 (0%)	7 (0.5%)
Stage I	77 (16.4%)	0 (0%)	29 (36.7%)	0 (0%)	0 (0%)	0 (0%)	0 (0%)	0 (0%)	0 (0%)	0 (0%)	0 (0%)	0 (0%)	0 (0%)	106 (7.6%)
Stage II	154 (32.8%)	0 (0%)	29 (36.7%)	0 (0%)	0 (0%)	0 (0%)	0 (0%)	0 (0%)	0 (0%)	0 (0%)	0 (0%)	0 (0%)	0 (0%)	183 (13.2%)
Stage III	170 (36.2%)	39 (100%)	20 (25.3%)	0 (0%)	0 (0%)	0 (0%)	0 (0%)	0 (0%)	0 (0%)	0 (0%)	0 (0%)	1 (100%)	0 (0%)	230 (16.6%)
Stage IV	23 (4.9%)	0(0%)	0 (0%)	0 (0%)	0 (0%)	0 (0%)	0 (0%)	0 (0%)	0 (0%)	0 (0%)	0 (0%)	0 (0%)	0 (0%)	23 (1.7%)
Missing	38 (8.1%)	0 (0%)	1 (1.3%)	198 (100%)	62 (100%)	65 (100%)	70 (100%)	49 (100%)	141 (100%)	19 (100%)	11 (100%)	0 (0%)	185 (100%)	839 (60.4%)
**stage**														
In-transit metastasis	0 (0%)	0 (0%)	0 (0%)	15 (7.6%)	0 (0%)	0 (0%)	0 (0%)	0 (0%)	0 (0%)	0 (0%)	0 (0%)	0 (0%)	0 (0%)	15 (1.1%)
Regional metastasis	0 (0%)	0 (0%)	0 (0%)	139 (70.2%)	0 (0%)	0 (0%)	0 (0%)	0 (0%)	0 (0%)	0 (0%)	0 (0%)	0 (0%)	0 (0%)	139 (10.0%)
Distant metastasis	0 (0%)	0 (0%)	0 (0%)	23 (11.6%)	0 (0%)	0 (0%)	0 (0%)	0 (0%)	0 (0%)	0 (0%)	0 (0%)	0 (0%)	0 (0%)	23 (1.7%)
Local recurrence	0 (0%)	0 (0%)	0 (0%)	11 (5.6%)	0 (0%)	0 (0%)	0 (0%)	0 (0%)	0 (0%)	0 (0%)	0 (0%)	0 (0%)	0 (0%)	11 (0.8%)
Missing	469 (100%)	39 (100%)	79 (100%)	10 (5.1%)	62 (100%)	65 (100%)	70 (100%)	49 (100%)	141 (100%)	19 (100%)	11 (100%)	1 (100%)	185 (100%)	1200 (86.5%)

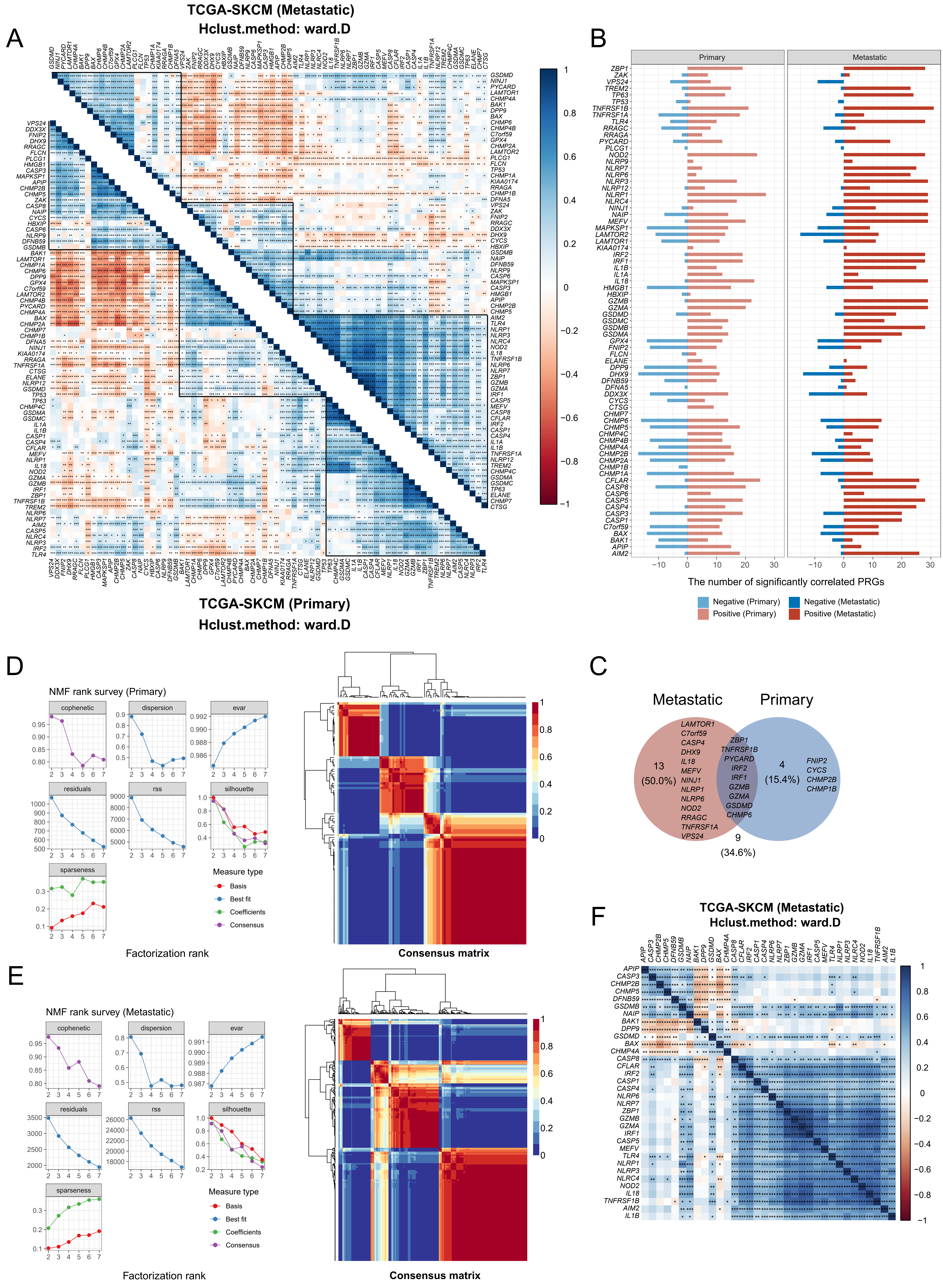

A
TCGA-SKCM (Metastatic)
Hclust.method: ward.D
TCGA-SKCM (Primary)
Hclust.method: ward.D
B
Primary
Metastatic
The number of significantly correlated PRGs
Negative (Primary)
Positive (Primary)
Negative (Metastatic)
Positive (Metastatic)
C
Metastatic
Primary
13
(50.0%)
LAMTOR1
C7orf59
CASP4
DHX9
IL18
MEFV
NINJ1
NLRP1
NLRP6
NOD2
RRAGC
TNFRSF1A
VPS24
ZBP1
TNFRSF1B
PYCARD
IRF2
IRF1
GZMB
GZMA
GSDMD
CHMP6
9
(34.6%)
4
(15.4%)
FNIP2
CYCS
CHMP2B
CHMP1B
D
NMF rank survey (Primary)
cophenetic
dispersion
evar
residuals
rss
silhouette
sparseness
Measure type
Basis
Best fit
Coefficients
Consensus
Factorization rank
Consensus matrix
E
NMF rank survey (Metastatic)
cophenetic
dispersion
evar
residuals
rss
silhouette
sparseness
Measure type
Basis
Best fit
Coefficients
Consensus
Factorization rank
Consensus matrix
F
TCGA-SKCM (Metastatic)
Hclust.method: ward.D

Fig. S1. Expression pattern and NMF clustering of PRGs in TCGA-SKCM. (**A**) Gene expression correlation of 74 pyroptosis-related genes in primary (bottom left) and metastatic (top right) samples. Blue and red indicate the magnitudes of correlations: blue, high; red, low. Significant points are labeled using *. Hierarchical clustering based on gene expression was used. (**B**) The bar graph shows the number of PRGs correlating positively or negatively with each PRG, with the primary tumor group on the left and metastatic tumor group on the right. The X axis represents the number of positively or negatively correlating PRGs, and the Y axis represents the PRGs. The positive and negative signs of the X axis coordinates represent positive or negative correlations. A significant correlation was considered when the absolute value of Pearson correlation coefficient was greater than 0.3 and p-value < 0.05. (**C**) Venn diagram showing PRGs correlating with GSDMD expression in primary and metastatic tumors. The number of genes in each part of the Venn diagram and their percentage in the overall population (26 genes) are indicated in the figure. Metastatic: 13 (50%); overlap: 9 (34.6%); primary: 4 (15.4%). (**D**-**E**) Optimal NMF clustering of mRNA expression profiles for primary (D) and metastatic (E) samples. The left image shows the parameters of NMF clustering, and the optimal rank was selected as the front point of the line segment with the minimum slope in the cophenetic plot. The image on the right is the consensus matrix drawn based on the features extracted by the NMF algorithm. (**F**) Gene expression correlation of 34 PRGs in metastatic samples. Both the horizontal and vertical axes are 34 PRGs. Blue and red indicate the magnitudes of the correlations: blue, high; red, low. Significant points are labeled using black asterisks.

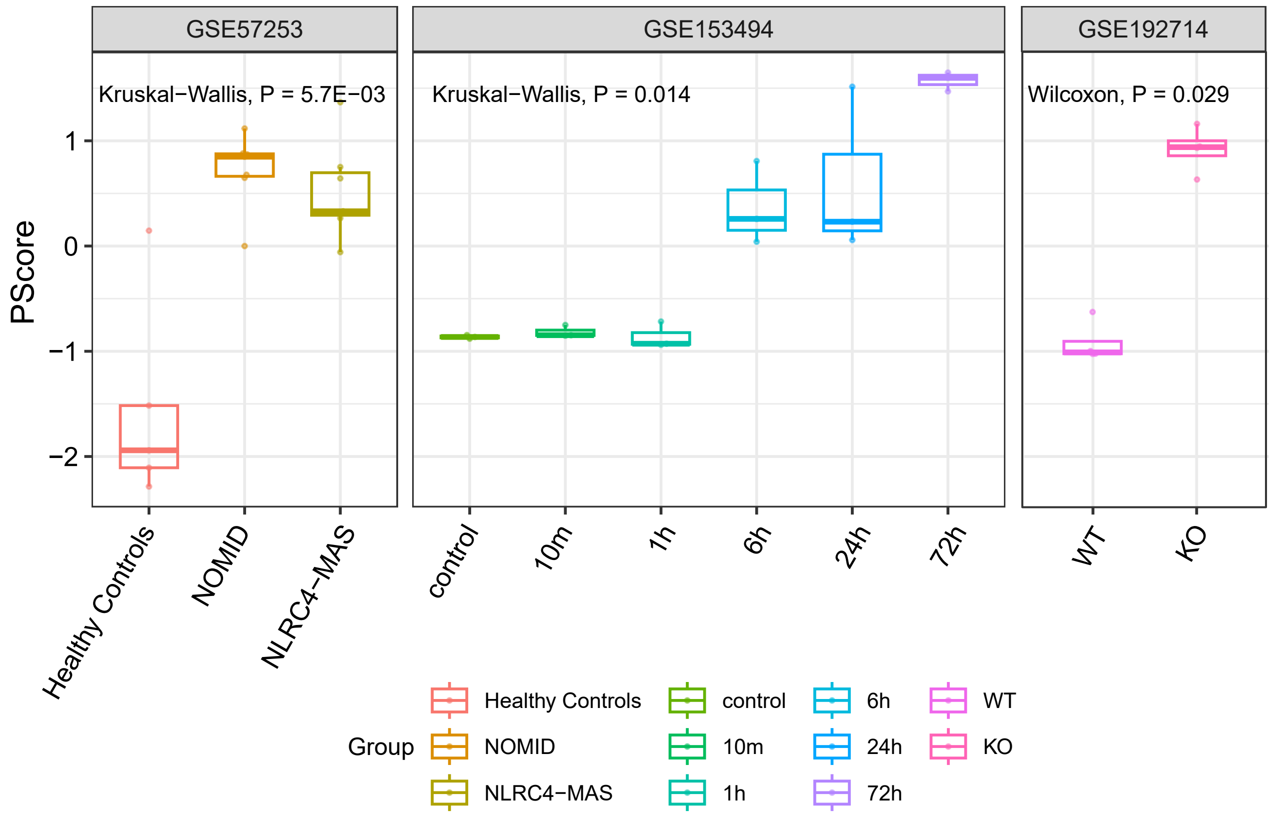

Fig. S2. Validation of the PScore model using 3 pyroptosis-related datasets. Pyroptosis-related datasets, including GSE57253, GSE153494 and GSE192714, were obtained from GEO, and PScore was calculated with significant genes using univariate Cox regression analysis. The x-axis represents the control group and experimental groups in the corresponding dataset. PScore was significantly higher in the experimental groups than the control group (GSE57253: left, Kruskal–Wallis, P = 5.7E-03; GSE153494: middle, Kruskal–Wallis, P = 0.014; GSE192714: right, Wilcoxon, P = 0.029). In GSE57253, NLRC4-macrophage activation syndrome (MAS) and neonatal-onset multisystem inflammatory disease (NOMID) are related to NLRC4 or NLRP3, respectively, and accompanied by IL-1β and IL-18 overproduction and increased pyroptosis. GSE153494 was used to describe the progression status of myocardial infarction associated with pyroptosis over time. Mll4 knockout elicits GSDMD-mediated pyroptosis in GSE192714.

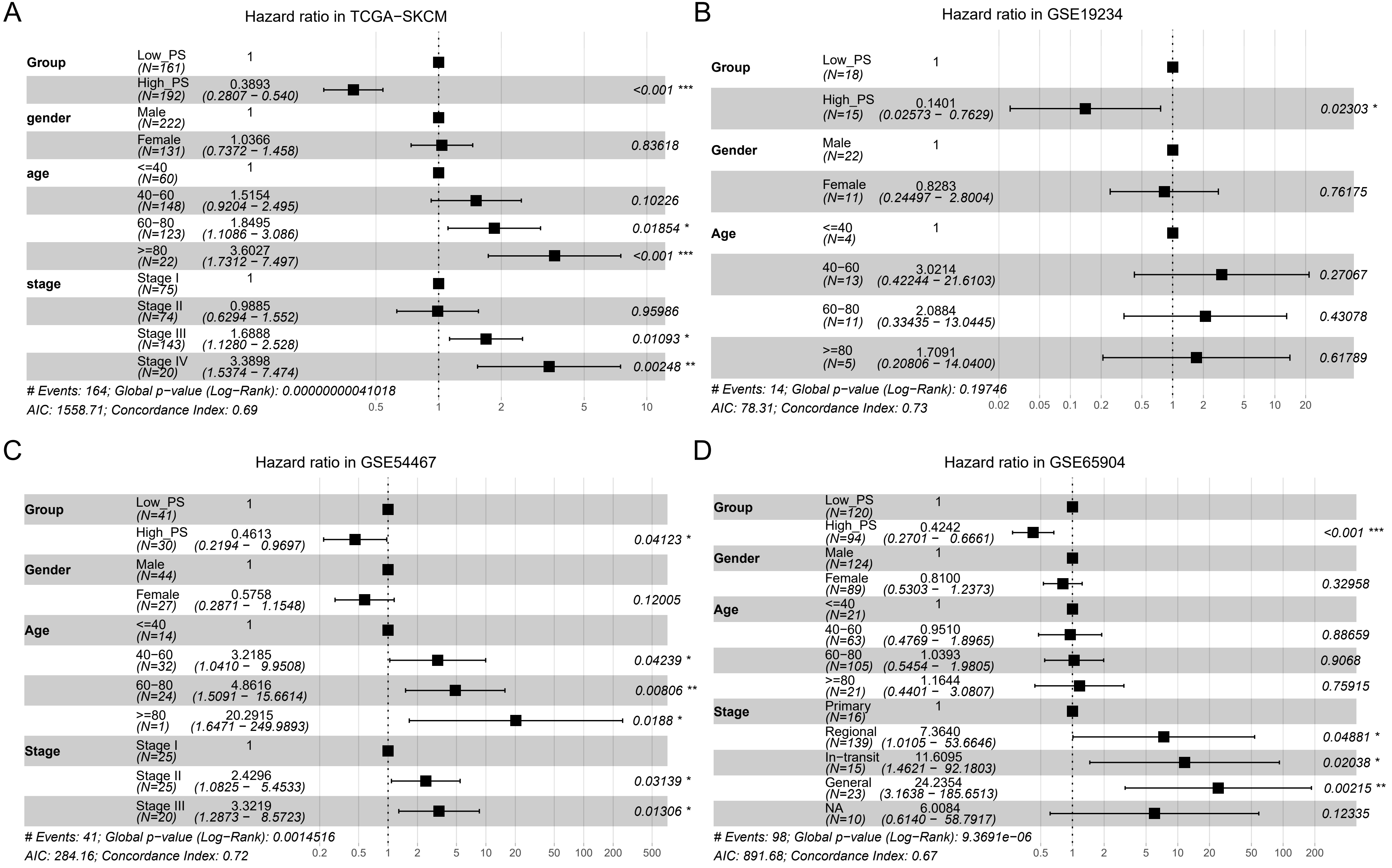

Fig. S3. Forest plot representation of multivariate Cox regression analyses using 4 datasets. Multivariate Cox regression analyses of PScore and 3 other variables, including sex, age and stage, in TCGA-SKCM (**A**) and 3 independent melanoma datasets, including GSE19234 (**B**), GSE54467 (**C**) and GSE65904 (**D**). Risk factors: HR > 1 and p-value <= 0.05; protective factors: HR < 1 and p-value <= 0.05; nonsignificant factors: p-value > 0.05.

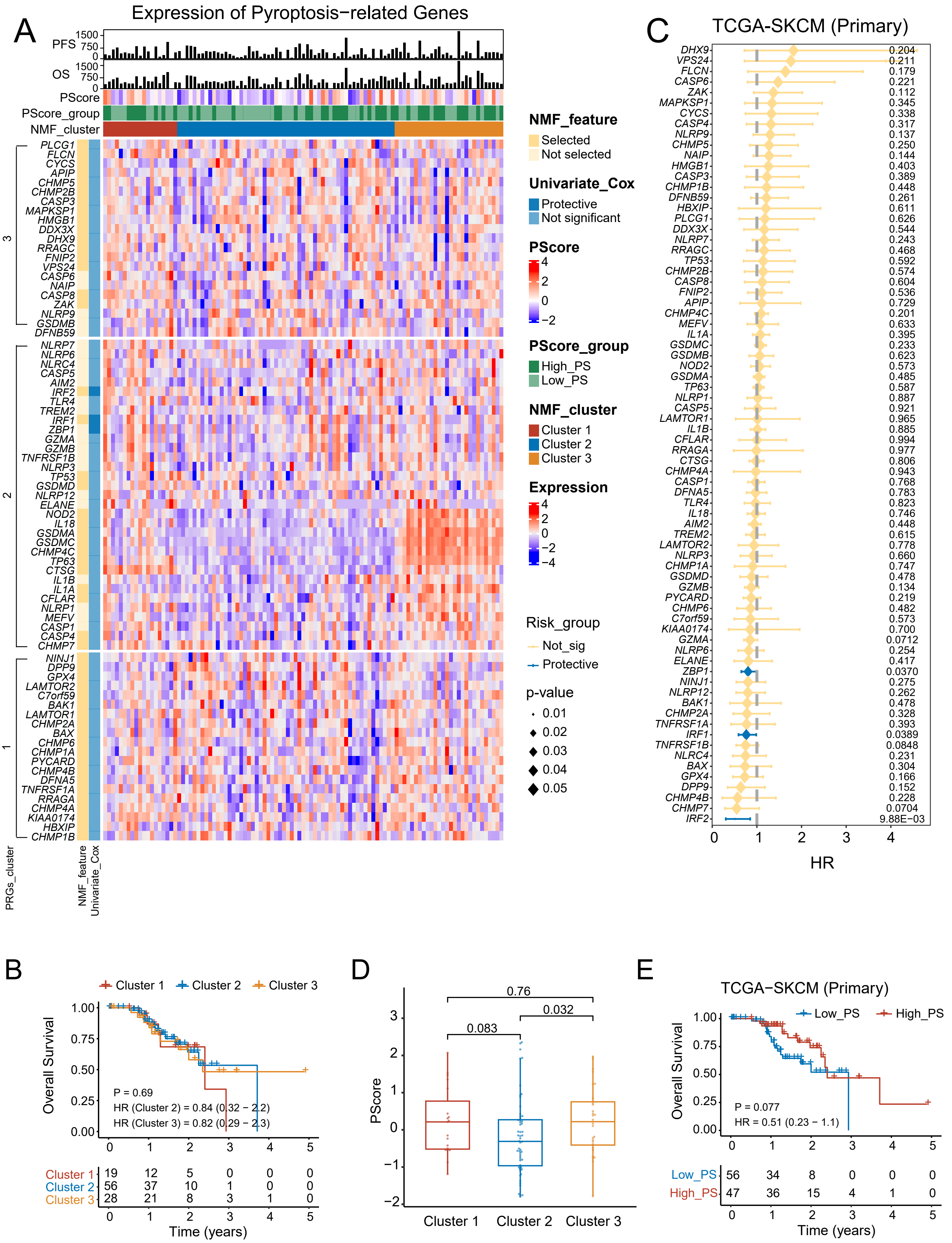
A
Expression of Pyroptosis−related Genes
PFS
OS
PScore
PScore_group
NMF_cluster
PRGs_cluster
NMF_feature
Univariate_Cox
NMF_feature
Selected
Not selected
Univariate_Cox
Protective
Not significant
PScore
PScore_group
High_PS
Low_PS
NMF_cluster
Cluster 1
Cluster 2
Cluster 3
Expression
Risk_group
Not_sig
Protective
p-value
0.01
0.02
0.03
0.04
0.05
C
TCGA-SKCM (Primary)
DHX9 0.204
VPS24 0.211
FLCN 0.179
CASP6 0.221
ZAK 0.112
MAPKSP1 0.345
CYCS 0.338
CASP4 0.317
NLRP9 0.137
CHMP5 0.250
NAIP 0.144
HMGB1 0.403
CASP3 0.389
CHMP1B 0.448
DFNB59 0.261
HBXIP 0.611
PLCG1 0.626
DDX3X 0.544
NLRP7 0.243
RRAGC 0.468
TP53 0.592
CHMP2B 0.574
CASP8 0.604
FNIP2 0.536
APIP 0.729
CHMP4C 0.201
MEFV 0.633
IL1A 0.395
GSDMC 0.233
GSDMB 0.623
NOD2 0.573
GSDMA 0.485
TP63 0.587
NLRP1 0.887
CASP5 0.921
LAMTOR1 0.965
IL1B 0.885
CFLAR 0.994
RRAGA 0.977
CTSG 0.806
CHMP4A 0.943
CASP1 0.768
DFNA5 0.783
TLR4 0.823
IL18 0.746
AIM2 0.448
TREM2 0.615
LAMTOR2 0.778
NLRP3 0.660
CHMP1A 0.747
GSDMD 0.478
GZMB 0.134
PYCARD 0.219
CHMP6 0.482
C7orf59 0.573
KIAA0174 0.700
GZMA 0.0712
NLRP6 0.254
ELANE 0.417
ZBP1 0.0370
NINJ1 0.275
NLRP12 0.262
BAK1 0.478
CHMP2A 0.328
TNFRSF1A 0.393
IRF1 0.0389
TNFRSF1B 0.0848
NLRC4 0.231
BAX 0.304
GPX4 0.166
DPP9 0.152
CHMP4B 0.228
CHMP7 0.0704
IRF2 9.88E-03
HR
B
Cluster 1
Cluster 2
Cluster 3
Overall Survival
P = 0.69
HR (Cluster 2) = 0.84 (0.32 − 2.2)
HR (Cluster 3) = 0.82 (0.29 − 2.3)
Cluster 1 19 12 5 0 0 0
Cluster 2 56 37 10 1 0 0
Cluster 3 28 21 8 3 1 0
Time (years)
D
PScore
0.76
0.032
0.083
Cluster 1
Cluster 2
Cluster 3
E
TCGA−SKCM (Primary)
Low_PS
High_PS
Overall Survival
P = 0.077
HR = 0.51 (0.23 − 1.1)
Low_PS 56 34 8 0 0 0
High_PS 47 36 15 4 1 0
Time (years)

Fig. S4. Clinical relevance of PRGs in primary cutaneous melanoma. (**A**) Heatmap showing expression of 74 PRGs in different NMF clusters or PScore groups of primary patients in the TCGA-SKCM cohort; red and blue denote high and low expression, respectively. The horizontal axis represents the individual patients, and the vertical axis represents the PRGs. PFS, OS and patient identities are shown above the heatmap. Light green and green are used to represent the 'Low_PS' and 'High_PS' groups. Red, blue and orange represent 'Cluster_1', 'Cluster_2' and 'Cluster_3' derived from NMF. The results of univariate Cox regression analysis of PRGs are annotated on the left, as are the PRGs selected as features during NMF. PRGs were divided into three subclusters (PRGs_cluster) using k-means clustering. (**B**) KM curves for OS in primary samples stratified by the NMF algorithm. The x-axis represents survival time (unit: year), and the y-axis represents OS rate. The colors of the KM curves represent different NMF-derived clusters. (**C**) Forest plot showing univariate Cox regression analysis of OS with 74 PRGs in primary melanoma patients. The x-axis represents the HR, and the y-axis represents the different PRGs. Yellow and blue indicate nonsignificant and protective genes, respectively. Protective genes: HR < 1 and p-value <= 0.05; nonsignificant genes: p-value > 0.05. (**D**) Comparison of PScore across NMF-derived clusters. The x-axis represents NMF-derived clusters, and the y-axis represents PScore. Colors correspond to the heatmap (A) and KM curve (B). (**E**) Kaplan–Meier curves for OS in primary samples stratified by PScore. The x-axis represents survival time (unit: year), and the y-axis represents OS rate. The colors of the KM curves represent different PScore groups.

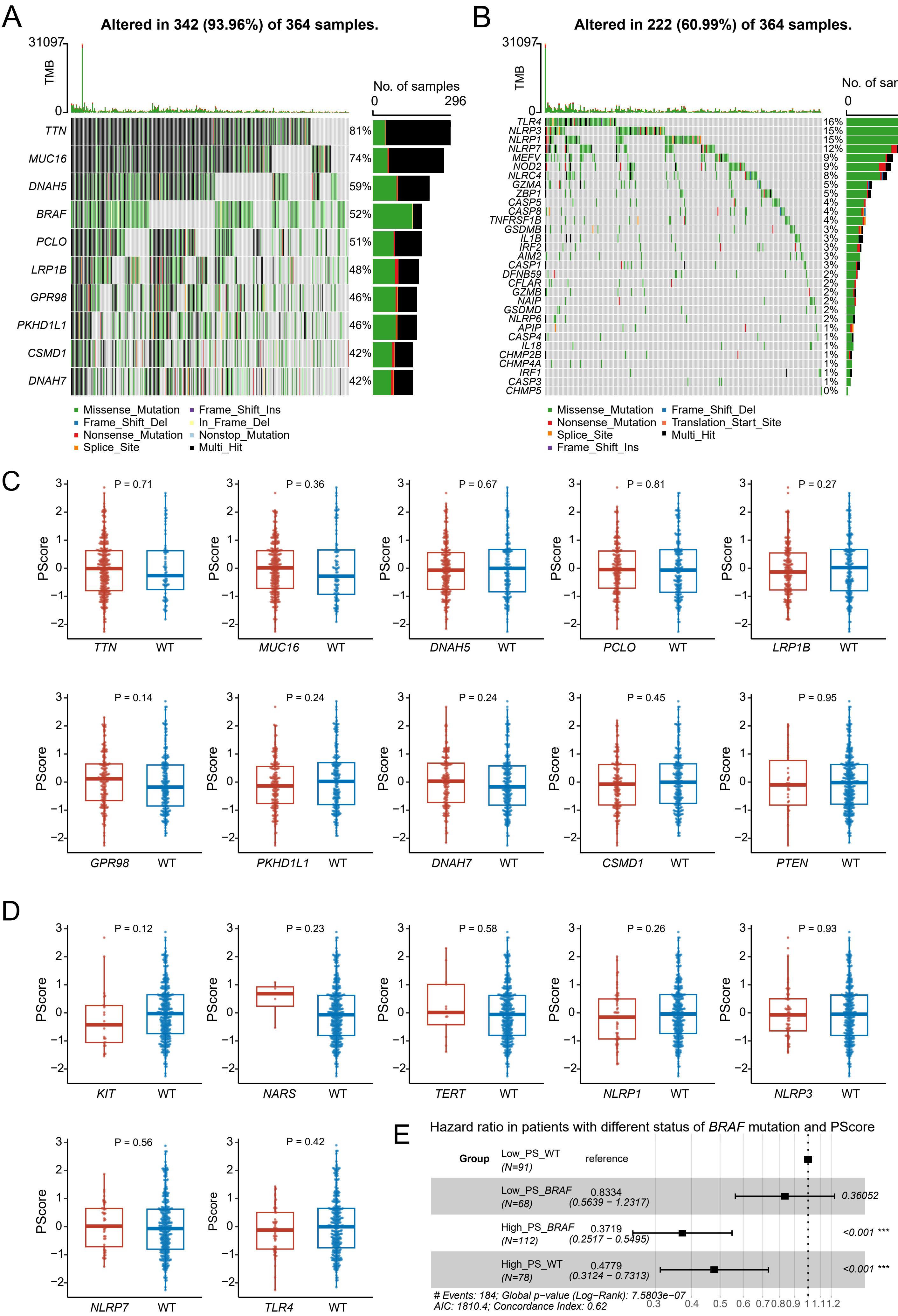
A
Altered in 342 (93.96%) of 364 samples.
31097
TMB
0
No. of samples
0
296
TTN 81%
MUC16 74%
DNAH5 59%
BRAF 52%
PCLO 51%
LRP1B 48%
GPR98 46%
PKHD1L1 46%
CSMD1 42%
DNAH7 42%
Missense_Mutation
Frame_Shift_Ins
Frame_Shift_Del
In_Frame_Del
Nonsense_Mutation
Nonstop_Mutation
Splice_Site
Multi_Hit
B
Altered in 222 (60.99%) of 364 samples.
31097
TMB
0
No. of samples
0
57
TLR4 16%
NLRP3 15%
NLRP1 15%
NLRP7 12%
MEFV 9%
NOD2 9%
NLRC4 8%
GZMA 5%
ZBP1 5%
CASP5 4%
CASP8 4%
TNFRSF1B 4%
GSDMB 3%
IL1B 3%
IRF2 3%
AIM2 3%
CASP1 3%
DFNB59 2%
CFLAR 2%
GZMB 2%
NAIP 2%
GSDMD 2%
NLRP6 2%
APIP 1%
CASP4 1%
IL18 1%
CHMP2B 1%
CHMP4A 1%
IRF1 1%
CASP3 1%
CHMP5 0%
Missense_Mutation
Frame_Shift_Del
Nonsense_Mutation
Translation_Start_Site
Splice_Site
Multi_Hit
Frame_Shift_Ins
C
PScore
P = 0.71
TTN WT
P = 0.36
MUC16 WT
P = 0.67
DNAH5 WT
P = 0.81
PCLO WT
P = 0.27
LRP1B WT
P = 0.14
GPR98 WT
P = 0.24
PKHD1L1 WT
P = 0.24
DNAH7 WT
P = 0.45
CSMD1 WT
P = 0.95
PTEN WT
D
P = 0.12
KIT WT
P = 0.23
NARS WT
P = 0.58
TERT WT
P = 0.26
NLRP1 WT
P = 0.93
NLRP3 WT
P = 0.56
NLRP7 WT
P = 0.42
TLR4 WT
E
Hazard ratio in patients with different status of BRAF mutation and PScore
Group
Low_PS_WT (N=91) reference
Low_PS_BRAF (N=68) 0.8334 (0.5639 − 1.2317) 0.36052
High_PS_BRAF (N=112) 0.3719 (0.2517 − 0.5495) <0.001 ***
High_PS_WT (N=78) 0.4779 (0.3124 − 0.7313) <0.001 ***
Events: 184; Global p-value (Log-Rank): 7.5803e-07
AIC: 1810.4; Concordance Index: 0.62
0.3 0.4 0.5 0.6 0.7 0.8 0.9 1 1.1 1.2

Fig. S5. Combining druggable mutations and PScore distinguishes the survival of metastatic *BRAF*-mutated melanoma patients. (**A-B**) Mutation landscapes of the top 10 high-frequency mutations and PRGs. The horizontal axis of the heatmap represents the patients, and the vertical axis represents the genes. (**C-D**) Boxplots show PScore in different mutation statuses in metastatic TCGA-SKCM. C: Top 10 high-frequency genes. D: Common therapeutic targets and top four PRGs. The x-axis represents mutation statuses, and the y-axis represents PScore. (**E**) Forest plot showing the HR of survival analysis related to Figure 2E. Risk factors: HR > 1 and p-value <= 0.05; protective factors: HR < 1 and p-value <= 0.05; nonsignificant factors: p-value > 0.05.

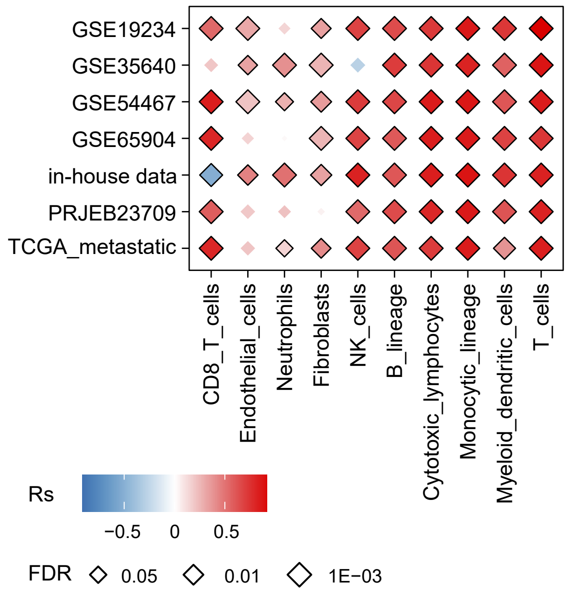

Fig. S6. Immune cell scores calculated with MCPcounter for seven datasets. Correlation of PScore with immune cell scores in multiple melanoma cohorts. The x-axis represents the type of infiltrating cells, and the y-axis represents the different datasets. 'TCGA_metastatic' refers to metastatic TCGA-SKCM patients. The color of the lattice represents the correlation coefficient. Significant points are labeled using black diamonds.

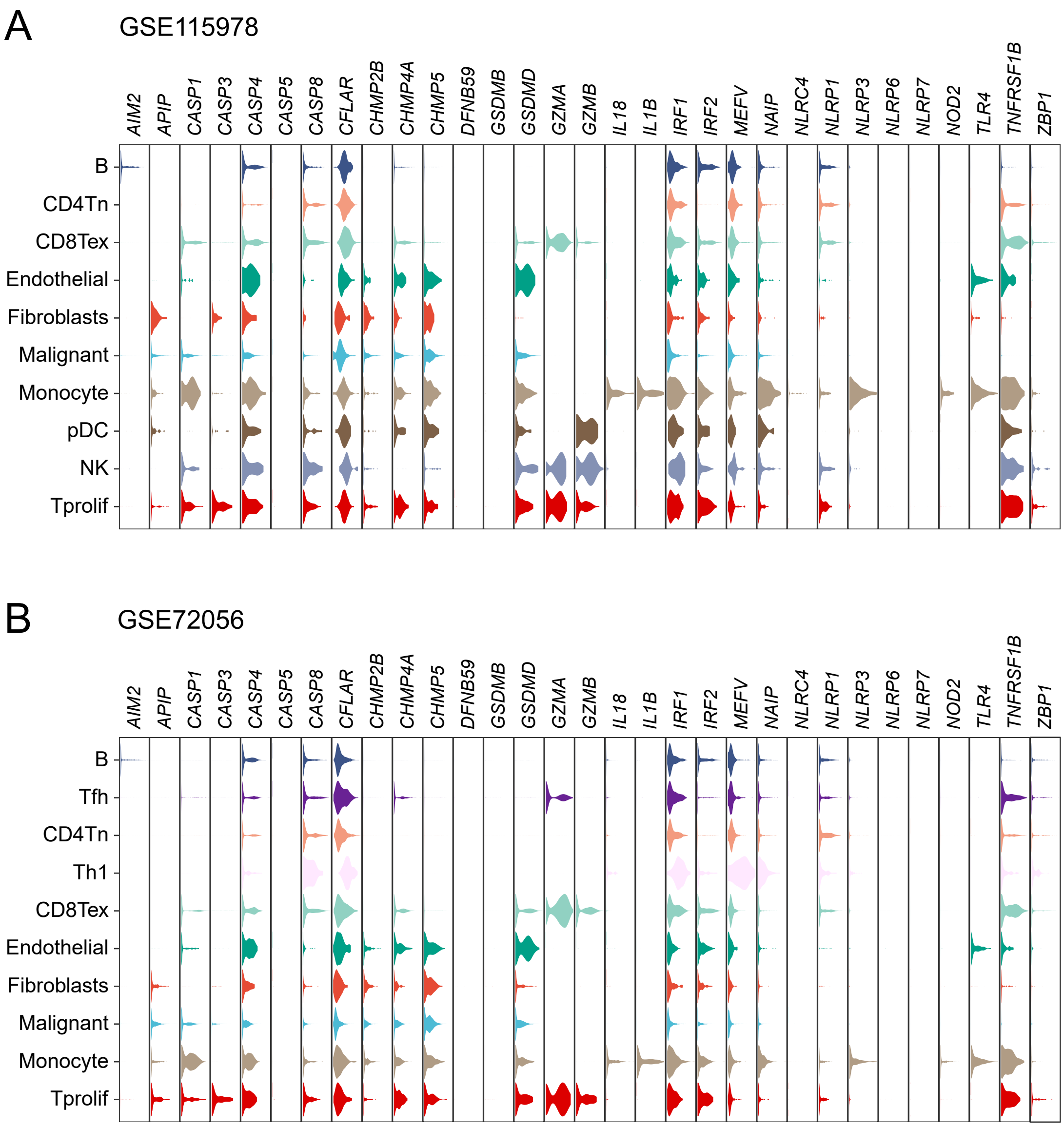

Fig. S7. Violin plots showing expression of the 31 genes used to calculate PScore corresponding to Figure 5B-E. (**A**) GSE115978, (**B**) GSE72056. The x-axis represents the 31 PRGs, and the y-axis represents the different cell subpopulations. The colors of the violin plot reflect cell types that are consistent with Figure 5B-E.

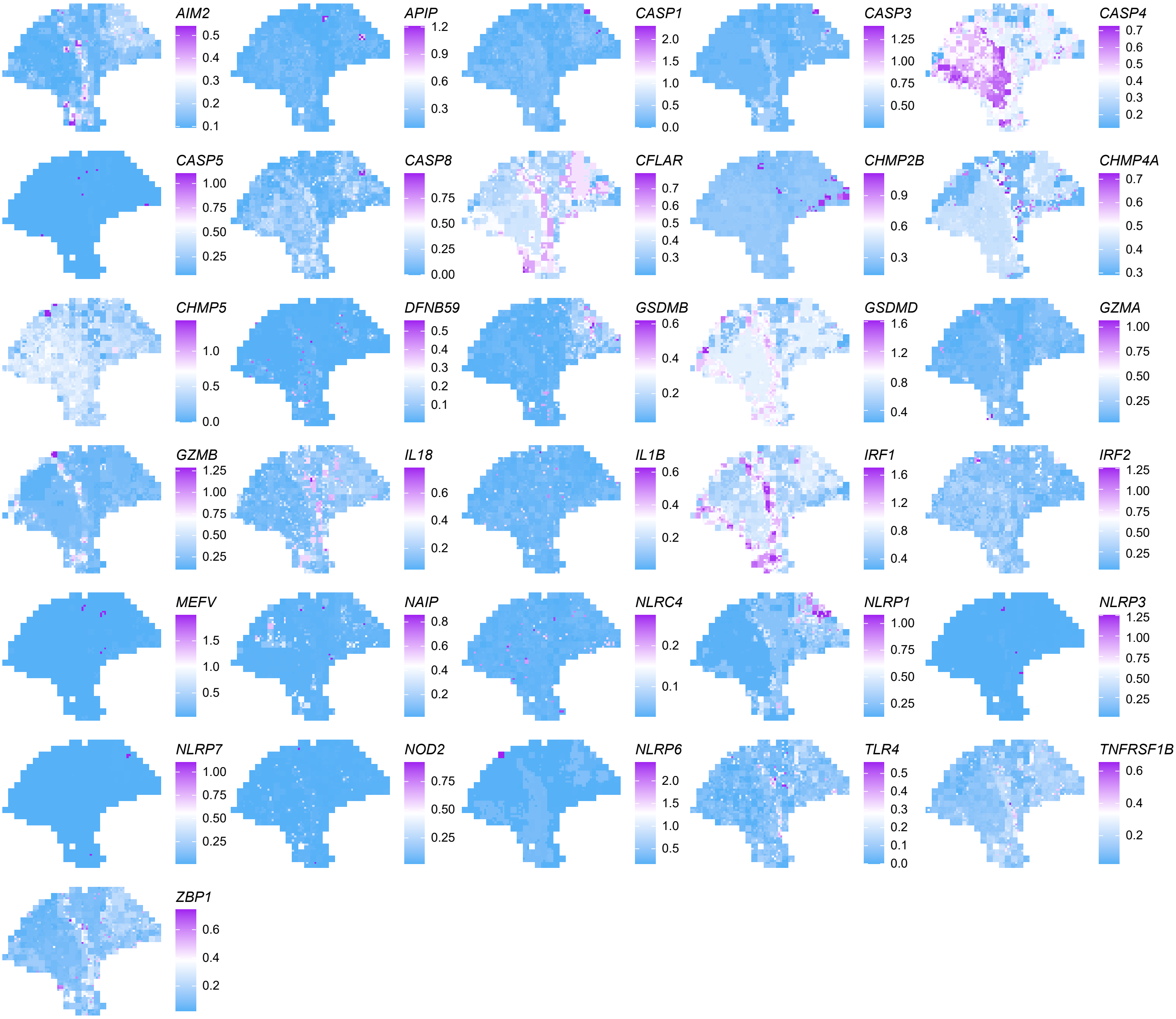

Fig. S8. Spatial expression of the 31 genes used to calculate PScore corresponding to Figure 5G-H. Blue, white and purple indicate the magnitudes of PRGs: blue, low; white, middle; purple, high. The corresponding cell annotations can be found in Figure 5F. These genes are sorted alphabetically.

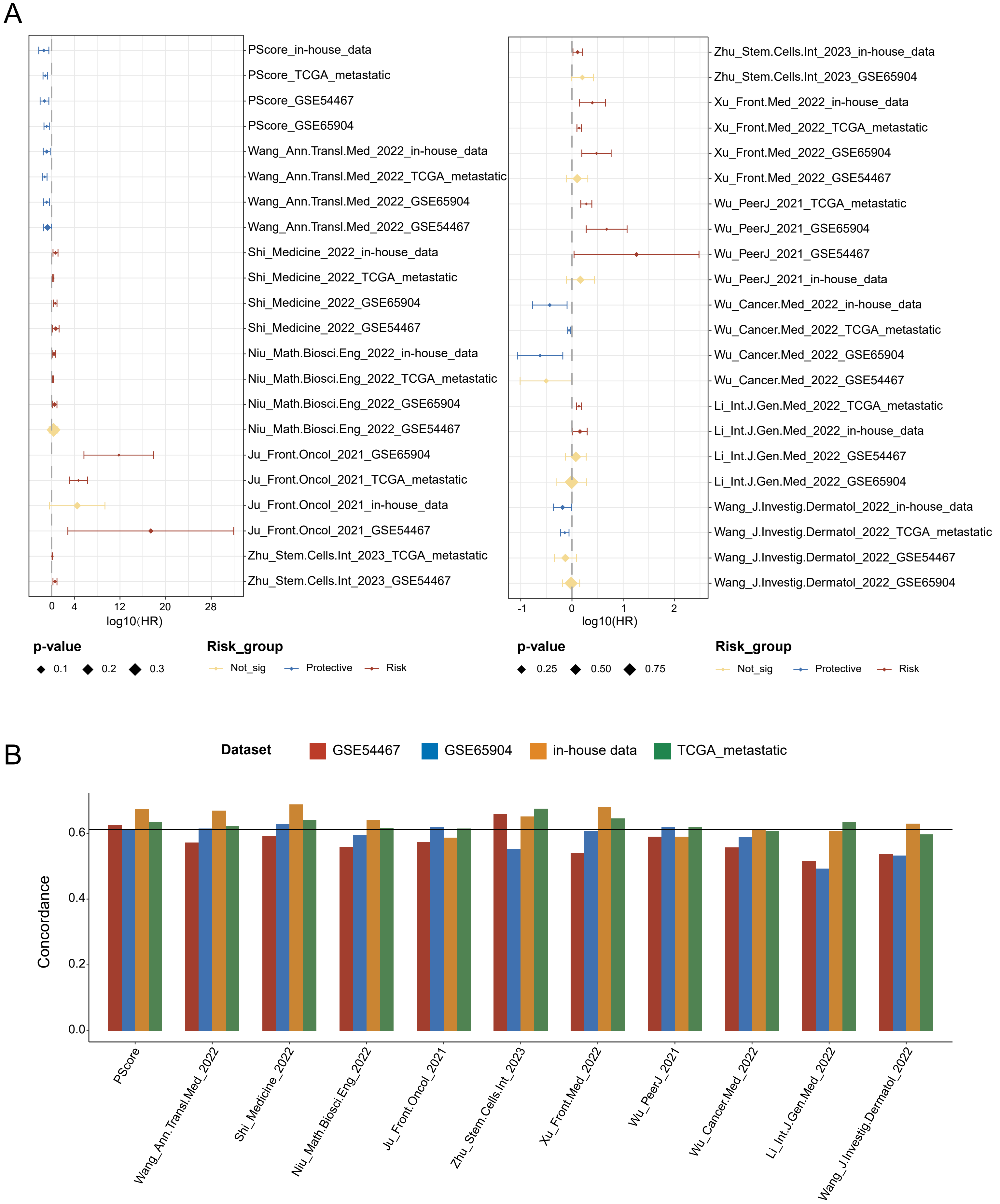

Fig. S9. Comparisons of the PScore model and other pyroptosis-related models. (**A**) Forest plot showing HR and confidence intervals of different methods in multiple datasets. The horizontal axis represents log10(HR), and the vertical axis represents different methods and datasets. (**B**) Bar chart showing the c-index of different methods in multiple datasets. The c-index performance of 11 methods, including ssGSEA using 31 protective PRGs (PScore) and 10 obtainable pyroptosis-related models, were compared across four datasets. The horizontal axis represents different methods, the vertical axis represents the c-index, and the colors of the bar graph represent different datasets.

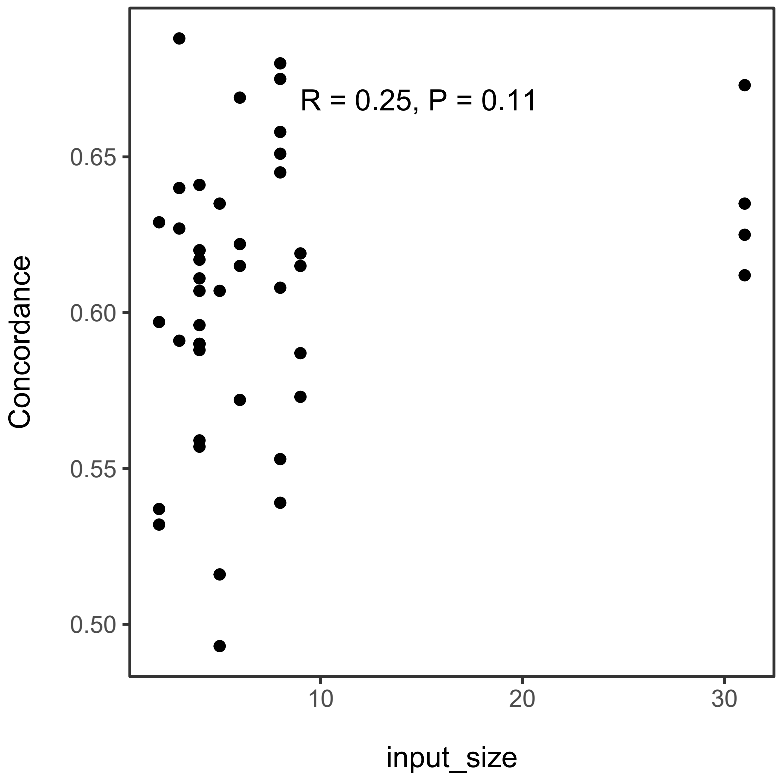

Fig. S10. Correlation between the number of input genes and the c-index. The correlation of gene input size for the 11 methods mentioned in Fig. S9 with concordance (c-index) across different datasets was analyzed. The x-axis represents the number of genes used in each method and the y-axis represents the c-index.
